# OsRALF26 Serves as an Endogenous Signal Recognised by XA21 to Promote Robust and Distal Resistance in Rice

**DOI:** 10.1111/pbi.70622

**Published:** 2026-03-11

**Authors:** Oh‐Kyu Kwon, A‐Ram Jeong, Chang‐Jin Park

**Affiliations:** ^1^ Department of Molecular Biology Sejong University Seoul South Korea; ^2^ Department of Bioresources Engineering Sejong University Seoul South Korea

**Keywords:** immunity, OsFLR1, RALF, rice, XA21, *Xanthomonas oryzae* pv. *oryzae*

## Abstract

Plant immune receptors detect both microbe‐derived and endogenous signals to activate defences. XA21, a rice immune receptor, confers strong race‐specific resistance to a subset of *Xanthomonas oryzae* pv. *oryzae* (*Xoo*) strains by recognising the microbial sulphated peptide RaxX. However, the molecular basis for the notably robust XA21‐mediated immune response has remained unclear. Here, we report that the small secreted peptide OsRALF26, previously identified as an *Oryza*‐specific ligand for FERONIA‐like receptor 1 (OsFLR1), is also directly perceived by XA21. Recognition of OsRALF26 by XA21 triggers a pronounced reactive oxygen species (ROS) burst, *pathogenesis‐related* (*PR*) gene induction, and enhanced resistance to *Xoo*. Notably, silencing *OsRALF26* leads to a spatially biased reduction in XA21‐mediated resistance, particularly in distal tissues. These findings identify OsRALF26 as a host‐derived ligand of XA21 that is required for full activation of XA21‐mediated immunity in distal tissues, consistent with a role for OsRALF26 in spatial propagation of XA21‐dependent defence. By integrating microbe‐derived and endogenous signals, XA21 exemplifies a versatile immune strategy in rice. This dual recognition may have arisen through the introgression of XA21, which unintentionally conferred OsRALF26 responsiveness—thereby reinforcing immune robustness in rice varieties.

## 1 Introduction

Plant immune receptors are broadly classified into membrane‐resident pattern recognition receptors (PRRs) and cytosolic nucleotide‐binding site leucine‐rich repeat proteins (NBS‐LRRs). PRRs recognise conserved microbe‐derived or endogenous (plant‐derived) signals—traditionally termed pathogen‐associated molecular patterns (PAMPs) and damage‐associated molecular patterns (DAMPs), respectively—to initiate pattern‐triggered immunity (De Lorenzo et al. 2018; Gomez‐Gomez and Boller 2000; Zipfel et al. 2006). In contrast, NBS‐LRRs detect specific pathogen‐delivered effectors, leading to robust defence responses and localised cell death (Jones and Dangl 2006; Wang et al. 2019).

Although this binary classification of PRRs and NBS‐LRRs has been useful, it has become increasingly insufficient as ongoing studies continue to reveal immune receptors that blur the boundaries between the two classes (Ercoli et al. 2022; Ngou et al. 2021; Ronald and Joe 2018). XA21, a rice LRR‐receptor‐like kinase (RLK), is a notable example of such complexity. Originally cloned from the wild rice species *Oryza longistaminata* , XA21 is often categorised as a PRR based on its structure and membrane localization (Ngou et al. 2022; Ronald et al. 1992; Song et al. 1995). However, it mediates unusually robust and race‐specific resistance against *Xanthomonas oryzae* pv. *oryzae* (*Xoo*)—features more commonly associated with NBS‐LRR receptors (da Silva et al. 2004; Ercoli et al. 2022). More than two decades after its cloning, XA21 was shown to recognise a microbial tyrosine‐sulphated peptide named RaxX (RaxX‐sY, required for activation of XA21‐mediated immunity X, tyrosine‐sulfated), secreted by certain *Xoo* strains (Ercoli et al. 2022; Pruitt et al. 2015). Unlike canonical PAMPs such as flg22 or elf18, which are derived from bacterial flagellin and elongation factor Tu and are broadly conserved across bacterial species (Felix et al. 1999; Gomez‐Gomez and Boller 2000; Zipfel et al. 2006), RaxX is restricted to a subset of *Xoo* strains and structurally mimics plant sulfated tyrosine (PSY) peptides that promote cell expansion (Amano et al. 2007; Pruitt et al. 2017, 2015). These atypical evolutionary features may explain XA21's race specificity, but they still raise key questions about the molecular mechanisms underlying its unusually strong immune activation. For clarity, we refer to XA21 hereafter as an ‘immune receptor’ and its ligands as either ‘microbe‐derived’ or ‘endogenous (plant‐derived)’, without strictly adhering to the conventional classification linking PRRs to PAMPs and DAMPs.

The rapid alkalinization factor (RALF) family of small, secreted peptides is well‐known for its role in inhibiting H^+^‐ATPase activity, swiftly alkalinizing the apoplast and arresting further cell extension through binding to *Catharanthus roseus* RLK1‐like (CrRLK1L) protein kinase family (Haruta et al. 2014; Kwon et al. 2024; Liu, Yeh, et al. 2024; Murphy and De Smet 2014; Pearce et al. 2001; Wu et al. 2007). The CrRLK1L family contains FERONIA (FER) and FER‐like receptors (FLRs), which feature an extracellular malectin‐like domain and a cytoplasmic kinase domain. However, recent studies reveal that certain RALF peptides are released upon pathogen attack and function as endogenous signals, triggering immune responses in line with their classification as canonical DAMPs (He et al. 2023; Kwon et al. 2024; Stegmann et al. 2017; Zhang et al. 2020). This dual functionality—spanning development and immune responses—reflects the remarkable signalling plasticity of RALF peptides in plant physiology.

OsRALF26 is transcriptionally induced by XA21 signalling and enhances disease resistance via its canonical receptor OsFLR1 (Kwon et al. 2024, 2025). Here, we investigated whether OsRALF26 also serves as a direct ligand of XA21 and how this interaction contributes to the strength and spatial dynamics of XA21‐mediated immunity to *Xoo*. Specifically, we examined whether OsRALF26, as an endogenous signal, is perceived by XA21 and how this interaction contributes to immune signalling both locally and in distal tissues. This study reveals how XA21 achieves its unusually robust and spatially coordinated immune responses.

## 2 Results

### 2.1 OsRALF26 Directly Interacts with XA21 In Vitro and In Planta

We previously reported that OsRALF26 promotes resistance to wild‐type *Xoo* in rice cultivar Kitaake via its canonical receptor OsFLR1 (Kwon et al. 2025, 2024) (Figure S1A–C, left panel). While extending this work, we found that exogenous application of the mature recombinant form of OsRALF26 (OsRALF26^mat^) further enhanced resistance in XA21‐overexpressing Kitaake (Ubi::XA21) plants (Figure S1A–C, right panel), suggesting a potential role for OsRALF26 in XA21‐mediated immunity. Consistent with this, OsRALF26^mat^ inhibited root elongation more strongly in Ubi::XA21 than in Kitaake (Figure S1D). In Kitaake, the enhanced resistance and root growth inhibition upon OsRALF26^mat^ treatment relative to mock or GFP controls can be explained by the presence of its canonical receptor OsFLR1. By contrast, the stronger responses observed in Ubi::XA21 plants suggested that XA21 might contribute to OsRALF26 perception, prompting us to test whether OsRALF26 physically interacts with XA21.

**FIGURE 1:**
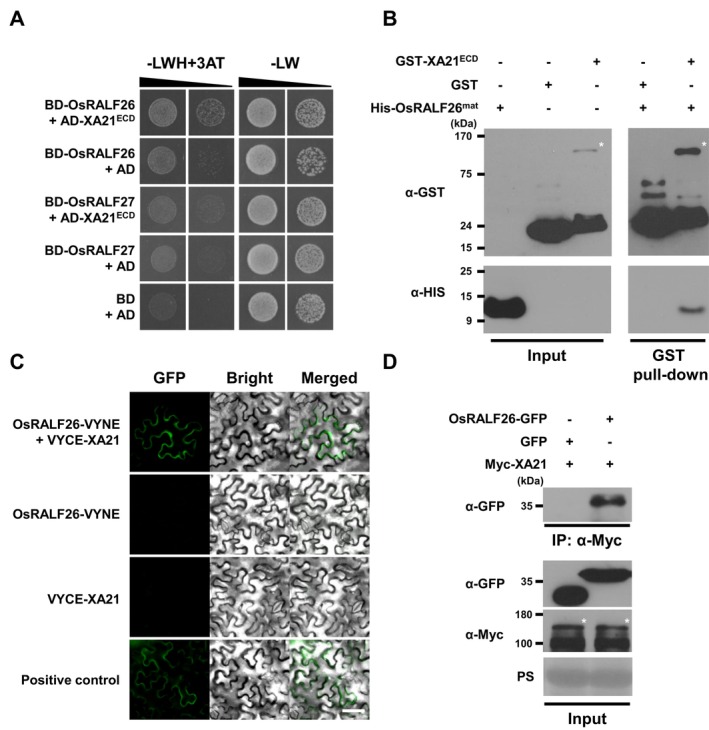
OsRALF26 directly interacts with the immune receptor XA21. (A) Interaction between OsRALF26 and the extracellular domain (ECD) of XA21 detected by yeast two‐hybrid (Y2H) assay. Yeast cells co‐expressing BD‐OsRALF26 or BD‐OsRALF27 with AD‐XA21^ECD^ were grown on selective dropout medium lacking Leu, Trp, and His (–LWH) supplemented with 15 mM 3‐aminotriazole (3‐AT). BD, DNA‐binding domain; AD, activation domain. (B) In vitro binding of OsRALF26 to XA21^ECD^ detected by pull‐down assay. Purified GST‐XA21^ECD^ or GST alone was incubated with His‐OsRALF26^mat^ and pulled down using glutathione Sepharose 4B beads. Proteins were detected by immunoblotting with anti‐His (α‐His) and anti‐GST (α‐GST) antibodies. His‐OsRALF26^mat^, ~9.95 kDa; GST‐XA21^ECD^, ~97.80 kDa; GST, ~28.42 kDa. Asterisk (*) indicates GST‐XA21^ECD^. (C) In planta interaction between OsRALF26 and XA21 visualised by bimolecular fluorescence complementation (BiFC) assay. OsRALF26‐VYNE and/or VYCE‐XA21 were transiently expressed in *Nicotiana benthamiana* leaves by agroinfiltration. YFP fluorescence was observed 2 days after infiltration using a fluorescence microscope. Scale bar, 50 μm. (D) Co‐immunoprecipitation (co‐IP) of OsRALF26 with XA21 in *N. benthamiana*. Leaves were co‐infiltrated with Myc‐XA21 and either OsRALF26‐GFP or GFP as a control. Co‐IP was performed 2 days after infiltration using anti‐Myc–conjugated agarose beads, and immunoblotting was performed with anti‐Myc (α‐Myc) and anti‐GFP (α‐GFP) antibodies. Myc‐XA21, ~110.83 kDa; OsRALF26^mat^‐GFP, ~36.77 kDa; GFP, ~26.79 kDa. Asterisk (*) indicates Myc‐XA21. All experiments were repeated three times with similar results.

A yeast two‐hybrid (Y2H) assay using the extracellular domain of XA21 (XA21^ECD^) revealed a specific interaction with OsRALF26 [DNA binding domain (BD)‐OsRALF26 + activation domain (AD)‐XA21^ECD^], but not with its closest homologue OsRALF27 (Kwon et al. 2025) (Figure 1A). This association was further confirmed by an in vitro pull‐down assay in which six‐histidine‐tagged mature OsRALF26 (His‐OsRALF26^mat^) co‐purified specifically with glutathione S‐transferase‐tagged XA21^ECD^ protein (GST‐XA21^ECD^) but not with GST alone (Figure 1B). We next tested for in planta interaction using bimolecular fluorescence complementation (BiFC) assay following agroinfiltration in *Nicotiana benthamiana*. Strong fluorescence was observed only when OsRALF26‐VYNE (N‐terminal fragment of the Venus protein) and VYCE (C‐terminal fragment)‐XA21 were co‐expressed as split‐YFP fusions, but not in controls (Figure 1C). This interaction was independently validated by in vivo co‐immunoprecipitation (co‐IP) assay from *N. benthamiana*, where C‐terminal GFP‐tagged OsRALF26 (OsRALF26‐GFP) specifically co‐purified with N‐terminal Myc‐tagged XA21 (Myc‐XA21) (Figure 1D). Together, these assays demonstrate that OsRALF26 directly interacts with XA21 both in vitro and in planta, providing a mechanistic basis for its involvement in XA21‐mediated immune signalling.

### 2.2 XA21 Functions as a Novel Immune Receptor for OsRALF26 in Rice

We next examined whether OsRALF26 directly regulates XA21‐mediates immune responses to in planta. To distinguish OsRALF26‐triggered immunity from those activated by XA21's canonical ligand RaxX, we employed a *Xoo* mutant strain *ΔraxST* (*Xoo* ^*ΔraxST*^), which is defective in sulfating RaxX and thus fails to activate XA21 (da Silva et al. 2004; Liu et al. 2019).

**FIGURE 2:**
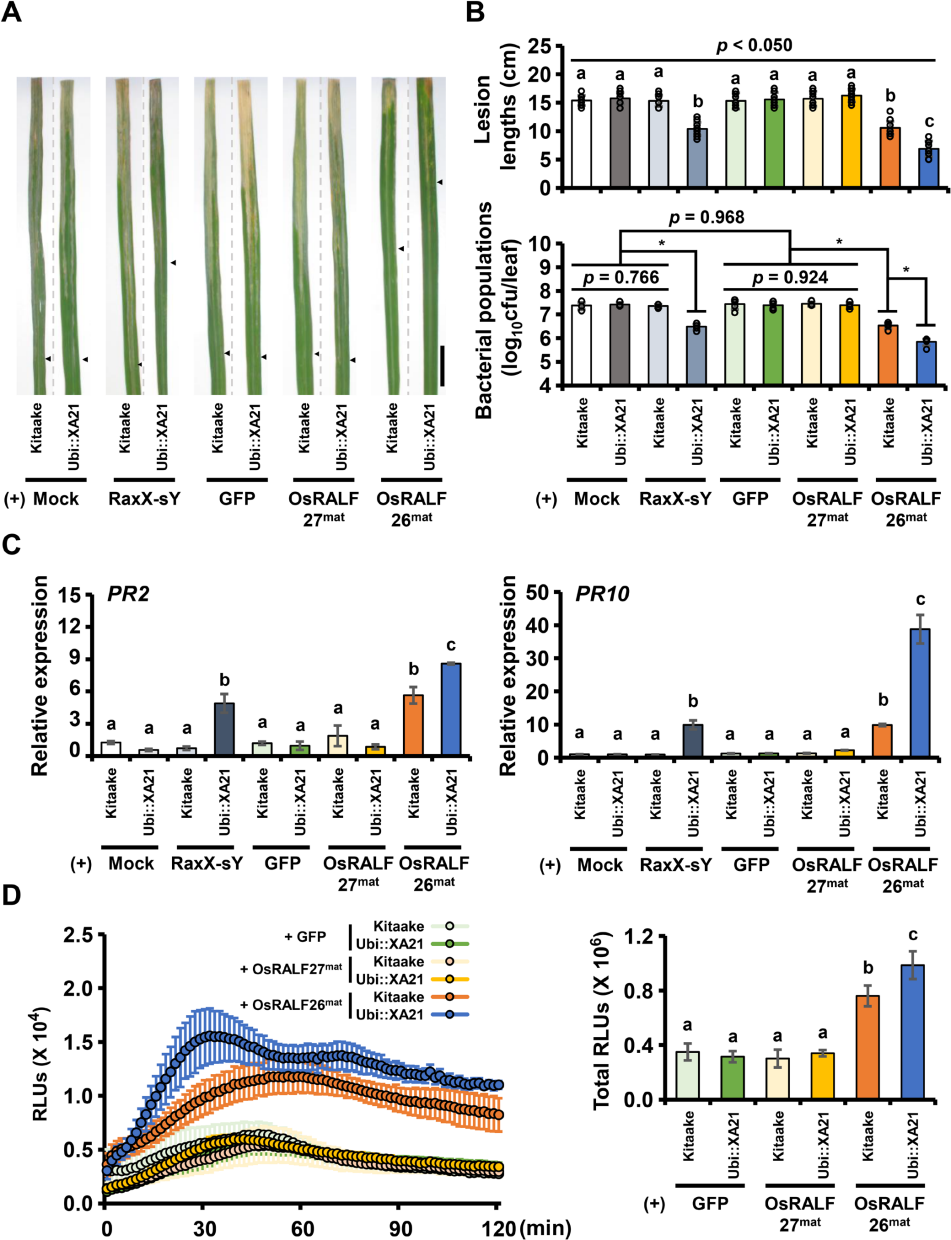
OsRALF26 enhances XA21‐mediated disease resistance in rice. (A) Representative leaf images of 6‐week‐old wild‐type Kitaake (lacking XA21) and Ubi::XA21 plants sprayed with 1 μM of RaxX‐sY, OsRALF26^mat^, OsRALF27^mat^ or GFP 1 day before inoculation with *Xoo* ^*ΔraxST*^. Leaves were photographed 14 days after inoculation. Arrowheads indicate the bottom boundary of the lesion on each leaf. Scale bar, 2 cm. (B) Lesion length (top) and bacterial population (bottom) measured 14 days after inoculation. Error bars represent SD of biological replicates (*n* = 8 for lesion length; *n* = 4 for bacterial population). (C) Expression of *PR2* and *PR10* in leaf segments of Kitaake and Ubi::XA21 plants treated with 1 μM RaxX‐sY, OsRALF26^mat^, OsRALF27^mat^ and GFP control. Error bars represent SD of technical replicates (*n* = 3). (D) ROS burst and cumulative ROS production over 120 min in leaf discs of 6‐week‐old Kitaake and Ubi::XA21 plants treated with 1 μM OsRALF26^mat^, OsRALF27^mat^ or GFP control. ROS was measured by a luminol‐based chemiluminescence assay and presented as accumulated relative luminescence units (RLUs). Error bars represent SD of biological replicates (*n* = 4). Different letters and asterisks represent significant differences (a–c *p* < 0.050 and *p < 0.050, one‐way ANOVA, Tukey's test). All experiments were repeated three times with similar results.

Treatment with OsRALF26^mat^ enhanced resistance to *Xoo* ^*ΔraxST*^ in both Kitaake and Ubi::XA21 plants, but the effect was significantly stronger in Ubi::XA21, as evidenced by reduced lesion lengths and bacterial populations (Figure 2A,B). In contrast, mock, GFP or OsRALF27^mat^ showed no difference in either genotype. The synthetic 21‐amino acid sulfated derivative (RaxX‐sY) served as a positive control (Kwon et al. 2024; Pruitt et al. 2015), inducing resistance in Ubi::XA21 but not in Kitaake. We next examined expression of defence marker genes. In both Kitaake and Ubi::XA21, OsRALF26^mat^ induced expression of *PR2* and *PR10*, but the induction levels were substantially higher in Ubi::XA21 (Figure 2C). As a positive control, RaxX‐sY specifically induced *PR2* and *PR10* in Ubi::XA21 but not in Kitaake. Similarly, OsRALF26^mat^ triggered a more robust ROS burst in Ubi::XA21 than in Kitaake (Figure 2D). Neither OsRALF27^mat^ nor GFP elicited a ROS burst in either background. Together, these results demonstrate that OsRALF26 activates typical immune responses more strongly in the presence of XA21, implicating XA21 as a potential immune receptor for OsRALF26.

### 2.3 XA21 Alone Is Sufficient to Mediate OsRALF26‐Triggered Immunity

To determine whether XA21 by itself can mediate OsRALF26‐triggered immunity, we used *N. benthamiana* as a heterologous system. This species does not respond to OsRALF26 under standard assay conditions (Kwon et al. 2025, 2024), suggesting the absence of endogenous receptors capable of recognising this peptide. This feature allowed us to assess XA21‐dependent responses to OsRALF26 without interference from OsRALF26‐triggered activation of FER/FLR in *N. benthamiana*. In rice, knocking out or silencing both OsFLR1 and OsFLR2 leads to severe developmental defects (Pu et al. 2017), making functional dissection in planta unfeasible.

**FIGURE 3:**
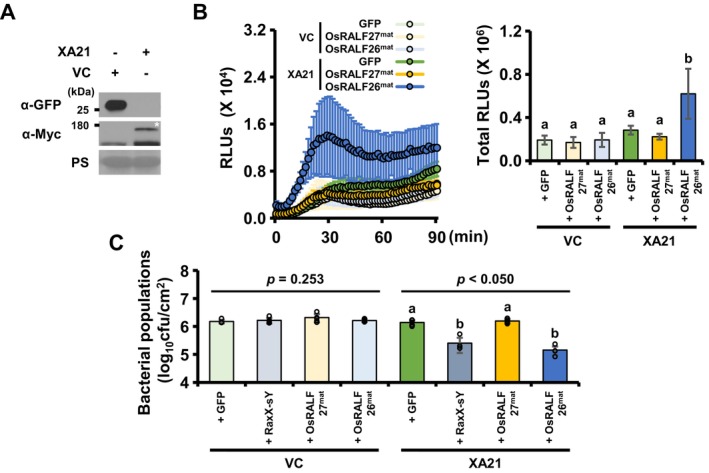
OsRALF26 activates XA21‐mediated immunity in *N. benthamiana* as a heterologous system. (A) Transient expression of Myc‐XA21 (XA21) or GFP (vector control, VC) in *Nicotiana benthamiana* leaves by agroinfiltration. Protein accumulation was analysed 2 days after infiltration by immunoblotting with anti‐Myc (α‐Myc) and anti‐GFP (α‐GFP) antibodies. Myc‐XA21, ~110.83 kDa; GFP, ~26.79 kDa. Asterisk (*) indicates Myc‐XA21. (B) ROS burst and cumulative ROS production over 90 min in leaf discs transiently expressing XA21 or VC, treated with 1 μM OsRALF26^mat^, OsRALF27^mat^ or GFP control. ROS was measured using a luminol‐based chemiluminescence assay and presented as accumulated RLUs. Error bars represent SD of biological replicates (*n* = 4). (C) Bacterial populations in transiently XA21‐ and VC‐expressing leaves treated with 1 μM RaxX‐sY, OsRALF26^mat^, OsRALF27^mat^ or GFP, then challenged with *Pseudomonas syringae* pv. *tomato* DC3000 (*Pst* DC3000) inoculation. Leaf samples were collected 3 days after inoculation, and bacterial loads (cfu/cm^2^) were quantified. Error bars represent SD of biological replicates (*n* = 5). Different letter represents significant differences (a, b *p* < 0.050, one‐way ANOVA, Tukey's test). All experiments were repeated three times with similar results.

We first confirmed that XA21 is functional in *N. benthamiana* by transiently expressing Myc‐XA21 and treating leaves with sulfated RaxX‐sY. As expected, this treatment triggered a robust ROS burst, whereas mock and inactive non‐sulfated RaxX‐Y treatments failed to elicit any response (Figure S2). Having validated this system, we treated *N. benthamiana* leaves transiently expressing XA21 (Nb‐XA21) or carrying the empty vector control (Nb‐VC) with OsRALF26^mat^, OsRALF27^mat^ or GFP (Figure 3A–C). In Nb‐VC leaves, none of the treatments triggered significant ROS production. Similarly, in Nb‐XA21 leaves, treatment with OsRALF27^mat^ or GFP failed to induce ROS. In contrast, OsRALF26^mat^ treatment in Nb‐XA21 leaves triggered a pronounced and rapid ROS burst, significantly stronger and faster than that in Nb‐VC leaves treated with OsRALF26^mat^ (Figure 3B). Cumulative ROS production over a 90‐min period showed that Nb‐XA21 leaves treated with OsRALF26^mat^ exhibited a dramatic (> 323%) increase in ROS compared to all controls, including Nb‐VC leaves treated with OsRALF26^mat^ and Nb‐XA21 leaves treated with OsRALF27^mat^ or GFP. We then tested whether the OsRALF26‐triggered ROS burst in Nb‐XA21 leaves leads to disease resistance against *Pseudomonas syringae* pv. *tomato* (*Pst*) DC3000. Pre‐treatment with OsRALF26^mat^ significantly reduced bacterial growth of *Pst* DC3000 in Nb‐XA21 leaves, but not in Nb‐VC leaves or in Nb‐XA21 leaves treated with OsRALF27^mat^ or GFP (Figure 3C). These results demonstrate that OsRALF26 functions as an endogenous signal when XA21 is present, confirming that the receptor is sufficient to mediate OsRALF26‐triggered immunity.

To assess whether XA21 kinase‐dependent early signalling is required for OsRALF26‐triggered immune responses, we employed a kinase‐dead XA21 variant carrying a Lys‐to‐Glu substitution at position 736 (XA21^K736E^), which has previously been shown to compromise XA21‐mediated signalling (Chen et al. 2010; Xu et al. 2006). Wild‐type XA21 and XA21^K736E^ were transiently expressed in *N. benthamiana*, and comparable protein accumulation was confirmed by immunoblot analysis (Figure S3A). We then compared early immune responses triggered by RaxX and OsRALF26 using ROS production as a functional readout. As expected, RaxX‐Y did not induce ROS in leaves expressing either XA21 or XA21^K736E^. In contrast, RaxX‐sY triggered a clear ROS burst in leaves expressing wild‐type XA21, whereas ROS production was not detectable in leaves expressing XA21^K736E^ (Figure S3B). Using the same system, treatment with OsRALF26^mat^ induced ROS accumulation in leaves expressing wild‐type XA21, but this response was largely abolished in leaves expressing XA21^K736E^ (Figure S3B). Together, these results indicate that OsRALF26‐induced ROS production depends on XA21 kinase activity, consistent with a requirement for an intact XA21 kinase domain in downstream immune signalling.

### 2.4 Enhanced Immune Signalling Through Dual Recognition of OsRALF26 by XA21 and OsFLR1

**FIGURE 4:**
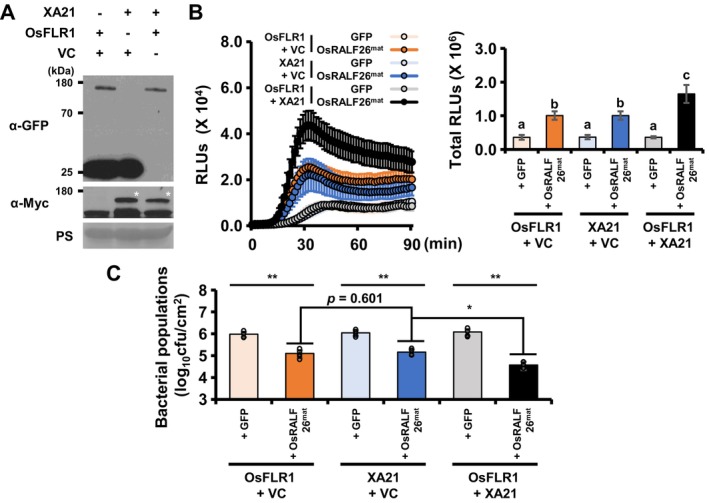
OsFLR1 enhances OsRALF26–XA21‐mediated immunity in *Nicotiana benthamiana*. (A) Transient co‐expression of OsFLR1‐GFP (OsFLR1), Myc‐XA21 (XA21) or GFP (VC) in *N. benthamiana* leaves. Protein accumulation was analysed 2 days after infiltration by immunoblotting with α‐Myc and α‐GFP antibodies. OsFLR1‐GFP, ~124.03 kDa; Myc‐XA21, ~110.83 kDa; GFP, ~26.79 kDa. Asterisk (*) indicates Myc‐XA21. (B) ROS burst and cumulative ROS production over 90 min in leaf discs co‐expressing OsFLR1 + VC, XA21 + VC, or OsFLR1 + XA21 after treatment with 1 μM OsRALF26^mat^ or GFP. ROS data are presented as accumulated RLUs. Error bars represent SD of biological replicates (*n* = 4). (C) Bacterial populations in leaves co‐expressing OsFLR1 + VC, XA21 + VC or OsFLR1 + XA21 treated with 1 μM OsRALF26^mat^ or GFP, then challenged with *Pst* DC3000 inoculation. Leaf samples were collected 3 days after inoculation, and bacterial loads. Error bars represent SD of biological replicates (*n* = 5). Different letters and asterisks represent significant differences (a–c *p* < 0.050, *p < 0.050 and **p < 0.001, one‐way ANOVA, Tukey's test). All experiments were repeated three times with similar results.

In rice, our results suggest that OsRALF26 is recognised not only by its canonical receptor OsFLR1 but also by XA21, raising the possibility that simultaneous recognition by both receptors may amplify immune responses. To test this hypothesis, we co‐expressed XA21 and OsFLR1 in *N. benthamiana* leaves and assessed both ROS production and pathogen resistance following OsRALF26^mat^ treatment. Myc‐XA21 and OsFLR1‐GFP were transiently expressed in *N. benthamiana* leaves either individually with an empty GFP control vector (Nb‐XA21 + Nb‐VC and Nb‐OsFLR1 + Nb‐VC) or together (Nb‐XA21 + Nb‐OsFLR1) (Figure 4A). As expected, GFP treatment alone did not elicit ROS production in any condition. OsRALF26^mat^ induced measurable ROS when either XA21 or OsFLR1 was expressed alone, but co‐expression of both receptors produced a substantially stronger and more sustained ROS burst (Figure 4B). This amplification was also reflected in pathogen resistance: pre‐treatment with OsRALF26^mat^ led to lower *Pst* DC3000 bacterial loads in Nb‐XA21 + Nb‐OsFLR1 compared to either receptor alone (Figure 4C). These findings imply that XA21 and OsFLR1 act independently or in a cooperative manner to mediate robust OsRALF26‐triggered immunity. This enhanced resistance observed in *N. benthamiana* raises the possibility that in rice, where both receptors are endogenously expressed, such dual recognition may contribute to the strength and robustness of OsRALF26‐mediated immune signalling.

### 2.5 OsRALF26 Is Required for XA21‐Mediated Immune Signalling in Distal Tissues

To examine the role of OsRALF26 in XA21‐mediated immunity, we generated a transgenic Kitaake line expressing *XA21* under its native promoter (Nat::XA21; Figure S4) and introduced OsRALF26‐RNAi constructs into this background (Figure S5A). Two independent RNAi lines (OsRALF26Ri/XA21 3‐2‐1 and 4‐2‐2) accumulated ~98% and ~99.6% fewer *OsRALF26* transcripts, corresponding to 0.018‐fold and 0.004‐fold of control levels, respectively. Notably, immunoblot analysis revealed comparable levels of XA21 protein accumulation between Nat::XA21 and OsRALF26‐RNAi plants (Figure S5B), and both RNAi lines exhibited normal growth and development under greenhouse conditions (Figure S5C).

**FIGURE 5:**
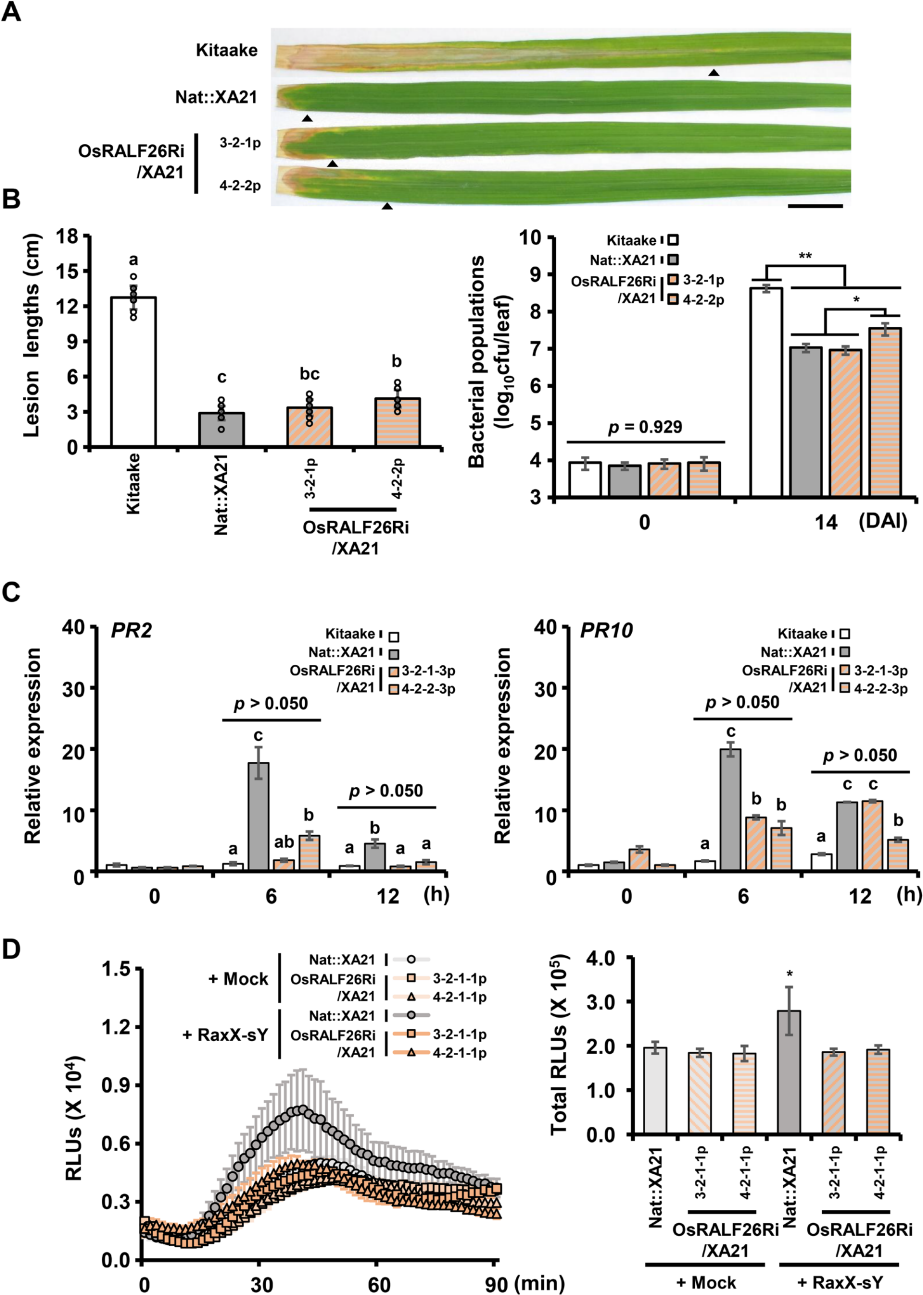
Silencing of OsRALF26 attenuates XA21‐triggered immune responses in rice. (A) Representative leaves of 8‐week‐old plants photographed 14 days after *Xoo* ^WT^ inoculation. From top to bottom: Kitaake (wild‐type), Nat::XA21 (transgenic Kitaake expressing XA21), and OsRALF26Ri/XA21 (transgenic Kitaake silencing *OsRALF26* in the Nat::XA21 background, T_3_; progeny of lines 3 and 4). Arrowheads indicate the bottom boundary of the lesion on each leaf. Scale bar, 2 cm. (B) Lesion length at 14 days (left) and bacterial population at 0 and 14 days (right) measured in 8‐week‐old rice leaves after *Xoo* ^WT^ inoculation. Error bars represent SD of biological replicates (*n* > 10 for lesion length; *n* = 3 for bacterial population). (C) Expression of *PR2* and *PR10* in Kitaake, Nat::XA21, and OsRALF26Ri/XA21 (T_4_; progeny of lines 3 and 4) after 1 μM RaxX‐sY treatment. Error bars represent SD of technical replicates (*n* = 3). (D) ROS burst and cumulative ROS production over 90 min in Nat::XA21 and OsRALF26Ri/XA21 (T_4_; progeny of lines 3 and 4) leaf discs treated with 1 μM RaxX‐sY or mock control. ROS was measured by luminol‐based chemiluminescence and expressed as accumulated RLUs. Error bars represent SD of biological replicates (*n* = 3). Different letters and asterisks represent significant differences (a–c *p* < 0.050, *p < 0.050 and **p < 0.001, one‐way ANOVA, Tukey's test). All experiments were repeated three times with similar results.

We first examined local immune responses at the site of infection by inoculating the leaves with wild‐type *Xoo* (*Xoo* ^WT^). In the strongly silenced OsRALF26Ri/XA21 line (4‐2‐2), lesion lengths and bacterial titers increased modestly compared to Nat::XA21, whereas the more weakly silenced line (3‐2‐1) showed no significant difference from Nat::XA21 at the statistical level (Figure 5A,B). However, upon RaxX‐sY treatment, allowing clearer observation of early immune signalling, both RNAi lines displayed significantly reduced *PR2* and *PR10* expression and attenuated ROS production (Figure 5C,D). These results suggest that OsRALF26 contributes to the early stages of XA21‐mediated immune activation.

As small secreted peptides, RALFs have been reported to signal in adjacent cells via receptors during growth, development, reproduction and defence (Chen et al. 2025; Kwon et al. 2024; Liu, Liu, et al. 2024; Zhou et al. 2024). To determine whether XA21‐mediated immunity extends into uninfected tissue, we tracked both bacterial movement and defence gene expression after inoculating *Xoo*. Using GFP‐labelling *Xoo* (*Xoo* ^GFP^), we found that bacteria spread only up to ~1 cm (R0_1 and R1_2) and did not progress beyond ~2 cm (R2_3) from the inoculation site within 3 days (Figure S6). In contrast, *PR10* expression, as expected, was detected at the inoculation site (T5), but notably, it was also observed in distal regions beyond 3 cm (R3_8), a zone not reached by *Xoo* (Figure S7). These observations suggest that, while *Xoo* cannot penetrate beyond ~2 cm 3 days after inoculation, an endogenous immune signal likely facilitates the spread of XA21‐triggered immune responses into distal tissue not yet reached by the pathogen.

**FIGURE 6:**
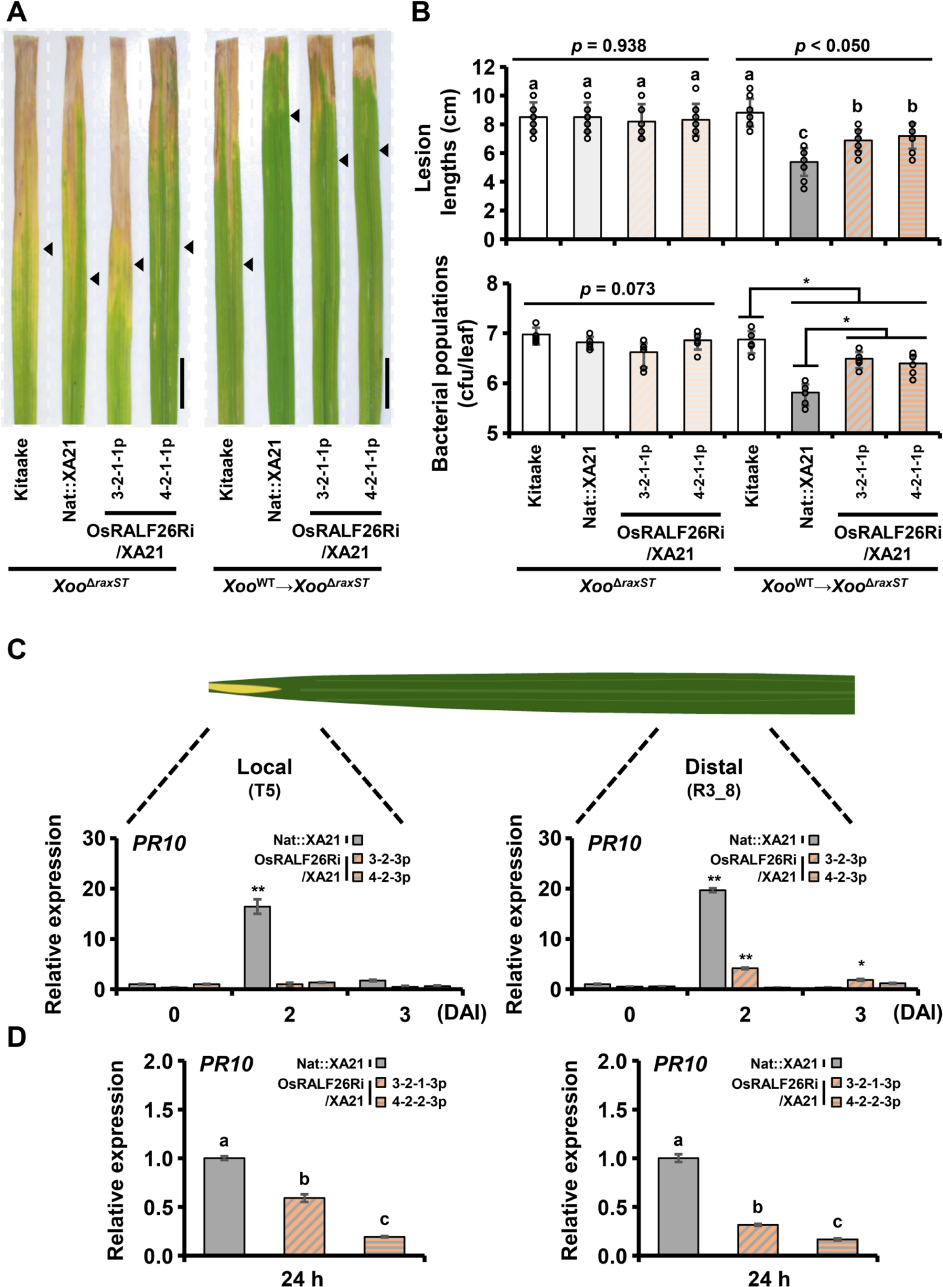
OsRALF26 contributes to distal activation of XA21‐mediated immunity in rice. (A) Representative leaves of 8‐week‐old plants photographed 14 days after *Xoo* ^*ΔraxST*^ or two‐step (*Xoo* ^WT^ → *Xoo* ^*ΔraxST*^) inoculation. From left to right: Kitaake, Nat::XA21, and OsRALF26Ri/XA21 (T_4_; progeny of lines 3 and 4). Arrowheads indicate the bottom boundary of the lesion on each leaf. Scale bar, 2 cm. (B) Lesion length (top) and bacterial population (bottom) measured in the same plants shown in (A). Error bars represent SD of biological replicates (*n* = 8 for lesion length; *n* = 5 for bacterial population). (C, D) Expression of *PR10* in Nat::XA21 and OsRALF26Ri/XA21 (T_3_ and T_4_; progeny of lines 3 and 4) leaves after *Xoo* ^WT^ inoculation (C) and RaxX‐sY treatment (D). T5 represents the 0–5 cm leaf segment directly treated with *Xoo* ^WT^ or 1 μM RaxX‐sY, and R3_8 represents the distal 3–8 cm segment located beyond the inoculated region. Error bars represent SD of technical replicates (*n* = 3). Different letters and asterisks represent significant differences (a‐c *p* < 0.050, *p < 0.050 and **p < 0.001, one‐way ANOVA, Tukey's test). All experiments were repeated three times with similar results.

To test whether OsRALF26 is required for the propagation of XA21‐dependent immunity to distal tissues, we developed a two‐step inoculation assay with *Xoo* ^WT^ and *Xoo* ^*ΔraxST*^ (Figure 6). Leaves were first inoculated with *Xoo* ^WT^ to activate XA21, and 3 days later, the proximal 0–3 cm segment was excised to eliminate residual bacteria. The immediately adjacent tissue was then inoculated with *Xoo* ^*ΔraxST*^, which cannot activate XA21 on its own. In Nat::XA21 plants, prior *Xoo* ^WT^ inoculation markedly enhanced resistance to *Xoo* ^*ΔraxST*^, resulting in shorter lesions and lower bacterial loads in the distal tissue (labelled as *Xoo* ^WT^ → *Xoo* ^*ΔraxST*^ in Figure 6A,B). In contrast, OsRALF26Ri/XA21 lines (3‐2–1‐1 and 4‐2–1‐1) exhibited significantly longer lesions and higher bacterial populations, indicating impaired distal immune signalling. As an additional control, direct inoculation of OsRALF26Ri/XA21 plants with *Xoo* ^*ΔraxST*^, without prior *Xoo* ^WT^ treatment resulted in susceptibility comparable to both Kitaake and Nat::XA21 (labelled as *Xoo* ^*ΔraxST*^ in Figure 6A,B). Consistently, *PR10* induction was markedly reduced in both the local (T5) and the distal (R3_8) regions of the OsRALF26Ri/XA21 lines compared to Nat::XA21 plants (Figure 6C,D). Taken together, these results indicate that OsRALF26 contributes to efficient propagation of RaxX‐triggered, XA21‐dependent immune signalling to distal tissues and support a model in which OsRALF26 functions as an endogenous signal that extends immune responses beyond the initial infection site.

## 3 Discussion

**FIGURE 7:**
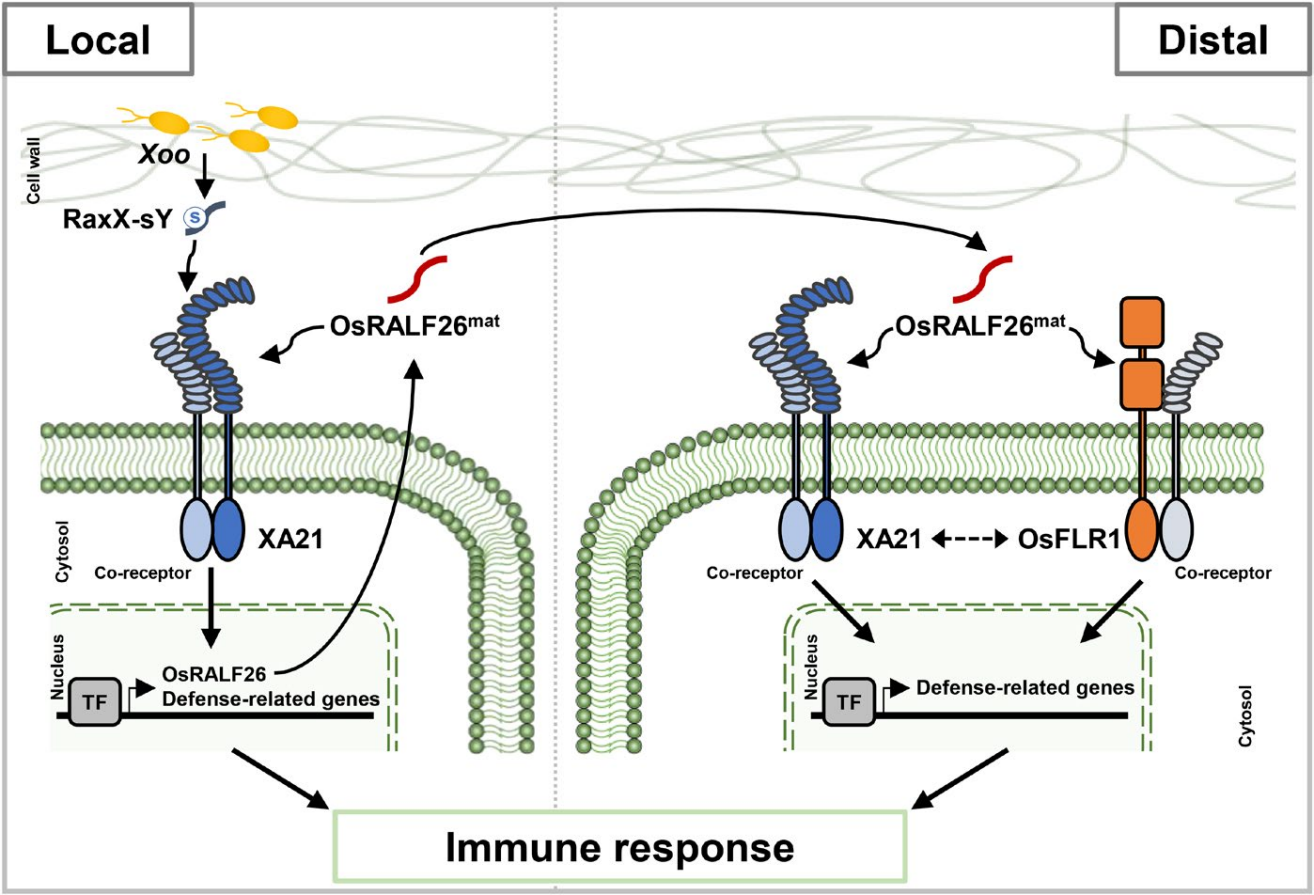
Proposed model for OsRALF26 in activating XA21‐mediated immunity in rice. OsRALF26 is transcriptionally induced by XA21 signalling and secreted into the apoplast, where it is directly recognised by XA21 to activate immune responses. In this model, XA21‐mediated immunity is triggered not only by the microbial ligand RaxX‐sY but also by the endogenous signal OsRALF26. Through this dual recognition, OsRALF26 contributes to resistance both locally at the infection site and in distal tissues. Together with the previously reported OsRALF26–OsFLR1 pathway (Kwon et al. 2024), the OsRALF26‐XA21 pathway reinforces the robustness and spatial propagation of immune response in rice.

In this study, we demonstrate that OsRALF26, previously known to activate immunity via the receptor OsFLR1 (Kwon et al. 2024), also interacts with XA21—an immune receptor classically known for recognising the microbial sulphated peptide RaxX from *Xoo* (Figure 7). This dual ligand recognition broadens the functional repertoire of XA21 and offers a mechanistic explanation for its robust and spatially extended immune activity.

Protein–protein interactions in plant immunity are generally highly specific. Nonetheless, members of the RALF peptide family can associate with structurally diverse partners. For example, Arabidopsis RALF1 (AtRALF1) not only interacts with its canonical receptor FER but also directly associates with the co‐receptor BAK1 to inhibit root cell expansion (Dressano et al. 2017). AtRALF23, also recognised by FER (Stegmann et al. 2017), binds the LORELEI‐like glycosylphosphatidylinositol‐anchored protein LLG1, which stabilises its interaction with FER (Xiao et al. 2019). AtRALF4/19 and AtRALF22/23 bind leucine‐rich repeat extensin (LRX) proteins in the cell wall to regulate pollen tube guidance and salt stress responses, respectively (Mecchia et al. 2017; Zhao et al. 2021). These findings underscore the structural versatility of RALFs in modulating diverse physiological and stress‐related responses (Liu, Yeh, et al. 2024; Murphy and De Smet 2014; Xiao et al. 2019; Zhao et al. 2021). However, to date, there are few—if any—reports of a single plant signalling peptide interacting with two structurally distinct receptors.

Here, we show that OsRALF26 activates immune responses via both OsFLR1 and XA21 in rice and *N. benthamiana*. Although we did not determine the binding affinity (Kd) of OsRALF26–XA21, the interaction is supported by multiple orthogonal assays (Y2H, pull‐down, BiFC and co‐IP) together with in planta genetic and physiological evidence. When both receptors are co‐expressed, OsRALF26‐induced responses are amplified beyond the level achieved by either receptor alone, suggesting additive or cooperative signalling. This dual recognition suggests notable structural plasticity, potentially mediated by an intrinsically disordered region (Figure S8), with the possible involvement of yet‐uncharacterized adaptable binding elements. XA21's ability to perceive two structurally, functionally, and evolutionarily distinct ligands—RaxX and OsRALF26—redefines it as a dual‐function immune receptor that integrates microbial perception with host‐derived signal amplification. This configuration forms a positive feedback loop: initial recognition of microbe‐derived RaxX induces OsRALF26, which in turn reinforces and spatially extends XA21 signalling. Such a mechanism could explain the unusually robust immune responses associated with XA21.

Although both ligands activate ROS production and *PR* gene expression via XA21, it remains unclear whether they engage identical receptor complexes or recruit distinct co‐receptor modules. Subtle differences in co‐receptor recruitment, signal kinetics, or subcellular localization may allow XA21 to tune downstream responses depending on the molecular origin and identity of the perceived signal. Dissecting the composition and dynamics of ligand‐specific XA21 complexes will be essential to determine whether microbe‐derived and endogenous signals converge or diverge in downstream signalling.

This dual recognition not only amplifies local immune responses but also supports the spatial propagation of XA21‐mediated defence. Reduced ROS and defence gene expression upon *OsRALF26* silencing does not proportionally translate into loss of XA21‐mediated disease resistance, likely reflecting differences in the spatial scale of immune responses. Although *Xoo* remains restricted to the local tissues during early infection, XA21‐expressing plants display elevated *PR* expression in distal leaf regions—a pattern significantly reduced in OsRALF26‐silenced lines. This indicates that OsRALF26 is required for full activation of XA21‐dependent immunity in distal tissues. As a small, secreted peptide previously reported to act beyond the producing cells (Kwon et al. 2024) and structurally stabilised by conserved disulphide bonds (Moussu et al. 2020), OsRALF26 is well positioned to contribute to immune activation beyond the initial infection site, together with its transcriptional induction by XA21 and direct interaction with it.

While we did not directly track OsRALF26 peptide movement in leaves, the spatial phenotype supports a model in which OsRALF26, or an OsRALF26‐dependent downstream signal, promotes defence activation in distal tissue. In distal regions, OsRALF26 may engage resident receptors such as OsFLR1 and XA21 to enhance immune responses to subsequent *Xoo* invasion, although whether these receptors act sequentially, in parallel, or with differing contributions remains to be determined. This interpretation is supported by our two‐step inoculation assay, where OsRALF26 was required for preemptive immune activation in tissues not yet exposed to the pathogen. The mechanism underlying this spatial effect remains to be clarified, and may be modulated by interactions with cell wall components such as pectins (Liu, Yeh, et al. 2024; Zhang et al. 2024). Together, these observations support a mechanism in which OsRALF26, induced locally by XA21, coordinates immune preparedness in distal tissues through dual‐receptor engagement.

To assess the evolutionary distribution of immune responsiveness to OsRALF26 and RaxX, we examined ROS production in diverse *Oryza* species using wild accessions obtained from IRRI (Figure S9 and Table S1). RaxX‐induced responses were detected only in *O. longistaminata* , whereas all tested species exhibited robust ROS induction upon OsRALF26 treatment. This indicates that OsRALF26 responsiveness is broadly conserved within the *Oryza* genus, in contrast to the restricted recognition of RaxX. XA21, originally cloned from *O. longistaminata* and subsequently introgressed into *O. sativa* for resistance breeding (Luo et al. 2012; Nguyen et al. 2018; Ronald et al. 1992; Song et al. 1995; Zhai et al. 2002), is well known for its role in race‐specific bacterial blight resistance. Our findings now suggest that OsRALF26—an endogenous peptide upregulated by XA21 activation and shown here to interact with XA21—may have been incorporated into this immune pathway during the introgression process. Such a co‐acquisition of OsRALF26 perception could enhance resistance robustness in XA21‐carrying rice cultivars by coupling microbial recognition with host‐derived signal amplification.

## 4 Experimental Procedures

### 4.1 Plant Materials and Growth Conditions

Rice ( *Oryza sativa* L. spp. japonica) variety Kitaake (lacking XA21), transgenic Kitaake lines, and wild rice were maintained in a greenhouse facility at Sejong University, Republic of Korea. *N. benthamiana* plants were grown in soil at 23°C under a 16/8 h (h) light/dark photoperiod. Eleven wild rice accessions used in this study were provided by the International Rice Research Institute (IRRI), Philippines (Table S1).

### 4.2 Plasmid Constructs

The construction of OsRALF26, OsRALF27, OsFLR1 and XA21 has been described previously (Chen et al. 2010; Kwon et al. 2025, 2024). For *OsRALF26* silencing, a 147‐nt cDNA fragment (nucleotides 101–247 of the *OsRALF26* CDS) was amplified from rice cDNA using specific primers (Table S2) and subcloned into pENTR using the pENTR/D‐TOPO kit (Invitrogen). This region was selected because of its low sequence similarity to other *OsRALF* paralogs. For silencing in rice, the partial OsRALF26 fragment (*OsRALF26Ri*) in pENTR was recombined into the pANDA silencing vector (Miki and Shimamoto 2004) using LR Clonase II enzyme mix (Invitrogen), generating *OsRALF26Ri*/pANDA. To express the XA21^ECD^ (residues 25–654), the corresponding cDNA was amplified with specific primers (Table S2) and subcloned into pENTR. The XA21^ECD^ in pENTR was recombined into the pDEST15 vector (Invitrogen), generating GST‐XA21^ECD^/pDEST15. For the Y2H assay, XA21^ECD^ in pENTR was recombined into the pDEST22 vector (Invitrogen), generating AD‐XA21^ECD^/pDEST22. For transient expression in *N. benthamiana*, Myc‐XA21 in pENTR was recombined into the pEarlyGate100 (pEG100) vector (Earley et al. 2006), generating Myc‐XA21/pEG100. To generate the kinase‐dead XA21 mutant, site‐directed mutagenesis was performed using the Q5 Site‐Directed Mutagenesis Kit (New England Biolabs) to substitute lysine 736 with glutamic acid (XA21^K736E^). The resulting Myc‐XA21^K736E^ construct was recombined into the pEG100 vector for transient expression assays. For the BiFC assay, OsRALF26 in pENTR was recombined into the pDEST‐^GW^VYNE vector (Gehl et al. 2009), generating OsRALF26‐VYNE/pDEST. In addition, the Myc tag in Myc‐XA21/pENTR was removed and replaced with VYCE‐HA using the restriction enzyme DraIII, and the modified construct was cloned into pEG100. All constructs were verified by Sanger sequencing (Macrogen).

### 4.3 Plant Transformation

Rice transformation was conducted as described previously (Chern et al. 2005). *Agrobacterium tumefaciens* strain LBA4404 harbouring *OsRALF26Ri*/pANDA was used to transform calli derived from transgenic Kitaake plants expressing XA21 under its native promoter (Nat::XA21). Transgenic plants were selected on hygromycin‐containing medium and verified by PCR using specific primers (Table S2) (Qiu et al. 2015).

### 4.4 RNA Extraction and RT‐qPCR Analysis

Total RNA was extracted from plant tissues after each treatment or inoculation using easy‐BLUE Total RNA Extraction Kit (iNtRON Biotechnology), following the manufacturer's instructions. RNA concentration was measured using the DS‐11 FX+ Nanodrop spectrophotometer (Denovix), and cDNA was synthesised from 1.5 to 2 μg of total RNA using random primers (Promega) and the M‐MLV reverse transcriptase (Promega). Expression of target genes was analysed by reverse transcription–quantitative PCR (RT‐qPCR) using a LightCycler 96 system (Roche) using GoTaq qPCR Master Mix kit (Promega) and specific primers (Table S2). Expression levels were normalised using *OsActin* as an internal reference gene.

### 4.5 Transient Gene Expression in *N. benthamiana*

For transient gene expression in *N. benthamiana* leaves, agroinfiltration was conducted as previously described (Kwon et al. 2024). *A. tumefaciens* strain GV3101 carrying each construct was cultured, harvested and resuspended in infiltration buffer to a final OD_600_ of 0.5 or 0.8. The bacterial suspension was incubated for 4 h at room temperature and then agroinfiltrated or co‐agroinfiltrated into 4‐ to 5‐week‐old *N. benthamiana* leaves using a 1 mL syringe. Transiently infiltrated leaves were returned to normal growth conditions for 2 days before analysis.

### 4.6 Purification of Recombinant Proteins

To purify recombinant proteins, His‐OsRALF26^mat^/pDEST17 (OsRALF26^mat^), His‐OsRALF27^mat^/pDEST17 (OsRALF27^mat^), His‐GFP/pDEST17 (GFP), GST‐XA21^ECD^/pDEST15 (GST‐XA21^ECD^) and GST/pGEX‐6P‐1 (Sigma‐Aldrich) were transformed into *E. coli* BL21 (DE3) cells (Thermo Fisher Scientific). Bacterial cultures were grown in LB medium at 37°C, and after reaching an OD_600_ of 0.5–0.8, the cells were induced for 3 h with 1 mM IPTG. Recombinant proteins were purified using Ni‐NTA agarose (Qiagen) and glutathione sepharose 4B (GE Healthcare), respectively, following the manufacturer's instructions.

### 4.7 Reactive Oxygen Species Measurement

ROS production assays were conducted as previously described (Kwon et al. 2024). For rice, leaf discs (4 mm in diameter) were collected from 6‐ to 8‐week‐old plants. Each disc was placed in a 96‐well white plate containing 100 μL sterile dH_2_O, one disc per well. After 16 h of recovery at room temperature in the dark, the water in each well was replaced with 100 μL of master mix solution [500 μM luminol L‐012 (FUJIFILM Wako) and 200 μg/mL horseradish peroxidase (HRP) (Sigma‐Aldrich), supplemented with 1 μM recombinant protein (OsRALF26^mat^, OsRALF27^mat^ or GFP) or 1 μM synthetic RaxX21‐sY (HVGGGDsYPPPGANPKHDPPPR) (ANYGEN)]. For agroinfiltrated *N. benthamiana*, leaf discs were collected 1 day after infiltration and placed in a 96‐well white plate containing sterile dH_2_O. The following day, the water was replaced with 2 mM MES‐KOH (pH 5.8) to mimic the plant apoplastic pH, and leaf discs were incubated for 3–4 h. After incubation, a 2× master mix solution was added to achieve final concentrations of 200 μM luminol L‐012, 50 μg/mL HRP, and either 1 μM recombinant protein or 1 μM synthetic RaxX21‐sY. Chemiluminescence was measured every second for 90–120 min using a Mithras LB 940 (Berthold). ROS production is presented as either progressive relative light unit (RLU) counts over time or as the integrated RLU counts.

### 4.8 Pathogen Inoculations

Wild‐type *X. oryzae* pv. *oryzae* strain PXO99 (*Xoo* ^WT^), *Xoo* lacking *raxST* (*Xoo* ^*ΔraxST*^) (Ronald 2014) and *Xoo* expressing GFP (*Xoo* ^GFP^) (Nozue et al. 2011) were grown on peptone sucrose agar (PSA) plates containing cephalexin for 3 days, then suspended in sterilised dH_2_O to an OD_600_ of 0.7, and used to inoculate 6‐ to 8‐week‐old Kitaake and transgenic plants using the scissors‐dip method (Chern et al. 2005; Kwon et al. 2024). For peptide‐induced resistance in rice, 1 μM of each recombinant protein (OsRALF26^mat^, OsRALF27^mat^ or GFP) or synthetic RaxX21‐sY was freshly prepared in 2 mM MES‐KOH (pH 5.8) with 0.05% Tween‐20 and sprayed onto rice plants. After 24 h, the pretreated plants were inoculated with *Xoo* ^WT^ or *Xoo* ^*ΔraxST*^. For two‐step *Xoo* inoculation, 3 days after *Xoo* ^WT^ inoculation, an additional *Xoo* ^*ΔraxST*^ inoculation was performed 3 cm behind the initial *Xoo* ^WT^ inoculation rice leaf site. *Pseudomonas syringae* pv. *tomato* (*Pst*) strain DC3000 was cultured in King's B medium supplemented with rifampicin and kanamycin at 28°C for 2 days, then suspended in 10 mM MgCl_2_ at an OD_600_ of 0.002 infiltration into leaves using a syringe. Pathogen inoculations on agroinfiltrated *N. benthamiana* leaves were performed as previously described with minor modifications (Buscaill et al. 2021). Two days after agroinfiltration, leaves were infiltrated with 1 μM of each recombinant protein or synthetic RaxX21‐sY in 2 mM MES‐KOH (pH 5.8), followed 24 h later by inoculation with *Pst* DC3000. Bacterial populations were quantified by plating on King's B agar supplemented with antibiotics and C.F.C. supplement (MBcell) to selectively allow the growth of *P. syringae* while inhibiting *A. tumefaciens* GV3101.

### 4.9 Yeast Two‐Hybrid Assay

A Y2H assay was performed using the ProQuest Two‐Hybrid System (Invitrogen) following the manufacturer's protocol. Yeast ( *Saccharomyces cerevisiae* ) strain MaV203 was separately transformed with bait plasmids (BD‐OsRALF26/pDEST32, BD‐OsRALF27/pDEST32 and BD‐empty/pDEST32) and prey plasmids (AD‐XA21^ECD^/pDEST22 and AD‐empty/pDEST22), and grown on synthetic defined medium lacking leucine and tryptophan (SD–LW) (Takara). Bait–prey combinations were produced by mating on SD–LW medium. Growth was tested by spotting 10 μL of each culture, adjusted to OD_600_ of 1.0 and 0.1, onto selective SD–LWH medium (lacking leucine, tryptophan, and histidine) (Takara). The medium was supplemented with 10–15 mM 3‐amino‐1,2,4‐triazole (3‐AT) and plates were incubated for 3 days at 30°C.

### 4.10 Bimolecular Fluorescence Complementation Assay and Microscopy

The *A. tumefaciens* strain GV3101 carrying the OsRALF26‐VYNE/pDEST or VYCE‐XA21/pDEST plasmid was used for BiFC. A suspension of GV3101 containing plasmids was mixed at a 1:1 ratio and infiltrated into *N. benthamiana* leaves. Rice leaves were collected 3 days after *Xoo* ^GFP^ inoculation to detect GFP signals. Fluorescence signals in *N. benthamiana* and rice leaves were observed using a fluorescence microscope Eclipse Ti (Nikon), fitted with an objective (40× or 200×). The filter sets used were C‐FL‐C FITC (excitation 465–495 nm) (Nikon) to detect GFP and YFP, respectively.

### 4.11 Western Blot Analysis

For western blot analysis, total proteins were extracted from ~0.1 g of homogenised tissue in 300 μL of denaturing extraction buffer (0.5× PBS with 5% SDS). The suspension was incubated for 5 min, boiled for 5 min, and centrifuged for 5 min to collect the supernatant. Protein concentration was determined using a microBCA protein assay kit (Biobasic). Total proteins (150–250 μg per well) were separated on 8%–15% SDS–PAGE gels and transferred to PVDF or nitrocellulose membranes (Bio‐Rad). For detection, primary antibodies [anti‐GFP (SC‐9996), anti‐Myc (SC‐47694), anti‐His (SC‐8036), and anti‐GST (SC‐53909); Santa Cruz Biotechnology] and an HRP‐conjugated secondary antibody [mouse IgG lambda binding protein (m‐IgGλ BP; SC‐516132‐CM; Santa Cruz Biotechnology)] were used. Immunoreactive bands were visualised using the SuperSignal West Pico Chemiluminescent Substrate (Thermo Fisher Scientific).

### 4.12 Pull‐Down Assays

For in vitro pull‐down assays, the purified recombinant His‐OsRALF26^mat^ (1 μM) and the glutathione sepharose 4B bound GST‐XA21^ECD^ or GST (100 μL) were mixed and incubated in 5 mL of interaction buffer [100 mM NaCl, 2.7 mM KCl, 8 mM NaH_2_PO_4_, 2 mM KH_2_PO_4_, 0.5% Triton X‐100, 1 mM PMSF, and cOmplete protease inhibitor (Roche) (pH 6.0)] for 3 h at 4°C. The protein complexes pulled down with glutathione sepharose 4B beads were washed three times with interaction buffer at 4°C, eluted by boiling in 40 μL SDS loading buffer for 10 min, and analysed by western blotting.

### 4.13 Co‐Immunoprecipitation Assay

Co‐IP assays were performed as previously described with minor modification (Kwon et al. 2024). Proteins were expressed in *N. benthamiana* leaves by co‐infiltrating *A. tumefaciens* strain GV3101 carrying each recombinant plasmid (OsRALF26/pEG103, GFP/pEG100 and Myc‐XA21/pEG100), and the leaves were harvested 48 h post‐infiltration. For interaction of XA21 and OsRALF26, proteins were extracted using co‐IP buffer (100 mM Tris–HCl pH 7.5, 150 mM NaCl, 5% glycerol, 1% TritonX‐100 containing 1 mM PMSF, 10 μM antipain, 10 μM aprotinin, 10 μM leupeptin, and cOmplete protease inhibitor). Protein extracts (2 g in 5 mL buffer) were incubated with 40 μL of anti‐Myc antibody–conjugated agarose (SC47694‐AC; Santa Cruz Biotechnology) for 3 h at 4°C. After washing three times with co‐IP buffer, the immune complexes were eluted by denaturation in 40 μL of SDS loading buffer and analysed by western blotting.

### 4.14 Statistical Analysis

Data are presented as mean and standard deviation (SD). Statistical significance was determined using one‐way ANOVA followed by Tukey's test. *p*‐values less than 0.050 or 0.001 were considered statistically significant.

### 4.15 Accession Numbers

Sequence data from this article can be found in the Rice Genome Annotation Project (https://rice.uga.edu/) and GenBank databases under the following identifiers: OsRALF26 (LOC_Os10g41980), OsRALF27 (LOC_Os10g41999), OsFLR1 (LOC_Os03g21540) and XA21 (U37133).

## Author Contributions

O.‐K.K. and C.‐J.P. conceived and designed the experiments. O.‐K.K. and A.‐R.J. performed the experiments and analysed the data. O.‐K.K. and C.‐J.P. wrote the manuscript. All authors read and approved the final manuscript.

## Funding

This work was supported by the NRF grant funded by the Korea government (MSIT) (RS‐2020‐NR047933 and RS‐2024‐00454908) and by the RISE programme through the Seoul RISE Center, funded by the MOE and the Seoul Metropolitan Government (2025‐RISE‐01‐019‐04).

## Conflicts of Interest

The authors declare no conflicts of interest.

## Supporting information


**Figure S1:** OsRALF26 enhances XA21‐mediated disease resistance and root growth inhibition in rice.
**Figure S2:** XA21‐mediated ROS burst is triggered by RaxX‐sY in *Nicotiana benthamiana*.
**Figure S3:** XA21 kinase domain is required for OsRALF26‐triggered ROS production in *Nicotiana benthamiana*.
**Figure S4:** Generation of transgenic rice plants expressing XA21 under its native promoter (Nat::XA21).
**Figure S5:** Generation of *OsRALF26*‐silenced transgenic rice in the Nat::XA21 background (OsRALF26Ri/XA21).
**Figure S6:** *Xoo* movement and XA21‐mediated defence response in distal rice leaf regions.
**Figure S7:** Induction of *PR10* in local and distal leaf regions after *Xoo* inoculation.
**Figure S8:**. Predicted intrinsically disordered regions of OsRALF26.
**Figure S9:** Evolutionary distribution of immune responsiveness to OsRALF26 and RaxX in wild rice species.
**Table S1:** List of *Oryza* accessions obtained from the International Rice Research Institute (IRRI).
**Table S2:** Primers used in this study.

## Data Availability

This study did not generate any new sequence data or accession numbers. All data supporting the findings of this work are available within the paper and its Supporting Information files.
