# Supplementary material for: OsRALF26 Serves as an Endogenous Signal Recognised by XA21 to Promote Robust and Distal Resistance in Rice
Source: Plant Biotechnol J. 2026 Mar 11;24(6):4159–73. doi: 10.1111/pbi.70622 (PMC13205699; doi:10.1111/pbi.70622)
Supplement: Supplementary file 1 — Figure S1: OsRALF26 enhances XA21‐mediated disease resistance and root growth inhibition in rice. Figure S2: XA21‐mediated ROS burst is triggered by RaxX‐sY in Nicotiana benthamiana. Figure S3: XA21 kinase domain is required for OsRALF26‐triggered ROS production in Nicotiana benthamiana. Figure S4: Generation of transgenic rice plants expressing XA21 under its native promoter (Nat::XA21). Figure S5: Generation of OsRALF26‐silenced transgenic rice in the Nat::XA21 background (OsRALF26Ri/XA21). Figure S6: Xoo movement and XA21‐mediated defence response in distal rice leaf regions. Figure S7: Induction of PR10 in local and distal leaf regions after Xoo inoculation. Figure S8:. Predicted intrinsically disordered regions of OsRALF26. Figure S9: Evolutionary distribution of immune responsiveness to OsRALF26 and RaxX in wild rice species. Table S1: List of Oryza accessions obtained from the International Rice Research Institute (IRRI). Table S2: Primers used in this study. [file PBI-24-4159-s001.docx]

**Supporting information
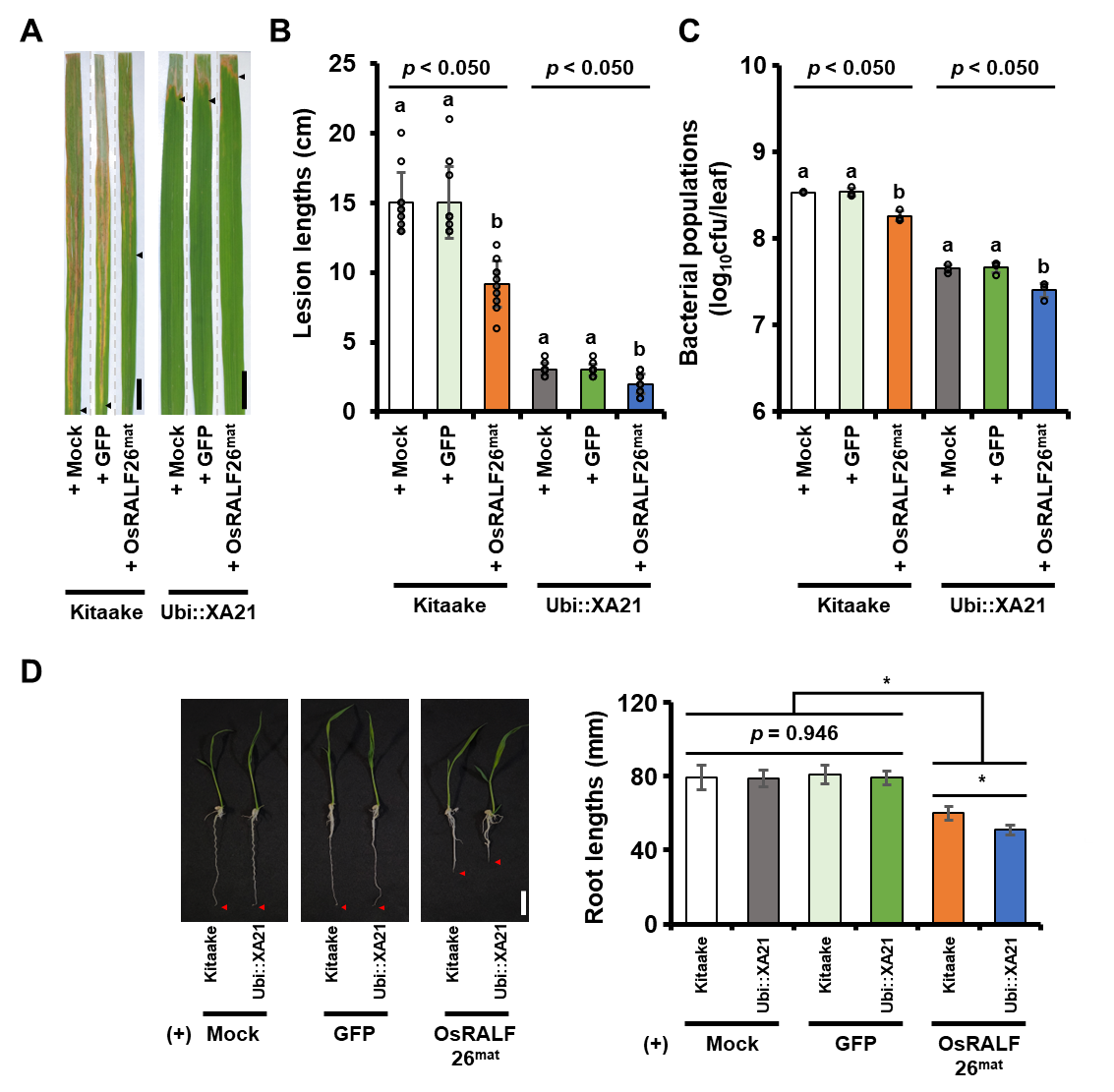
**

**Figure S1. OsRALF26 enhances XA21-mediated disease resistance and root growth inhibition in rice. (A)** Representative leaf images of rice plants (Kitaake and Ubi::XA21) sprayed with 1 μM OsRALF26^mat^ or GFP control 1 day before inoculation with *Xoo*^WT^. Leaves were photographed 14 days after inoculation. Arrowheads indicate the bottom boundary of the lesion on each leaf. Scale bar, 2 cm. **(B,C)** Lesion length **(B)** and bacterial population **(C)** measured in the same plants shown in **(A)**. Error bars represent SD of biological replicates (*n* = 10 for lesion length; *n* = 3 for bacterial population). **(D)** Root length of 5-day-old seedlings grown on 1/2 MS medium supplemented with 1 μM OsRALF26^mat^, or GFP. Representative seedlings and quantification are shown. Error bars represent SD of biological replicates (*n* = 4). Different letters and asterisks represent significant differences (a-b *p* < 0.050 and **p* < 0.050, one-way ANOVA, Tukey’s test). All experiments were repeated three times with similar results.

**
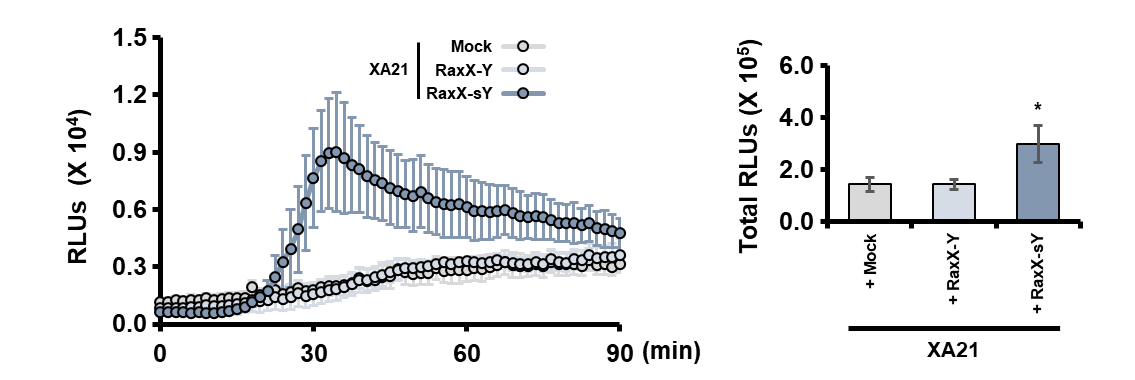
**

**Figure S2. XA21-mediated ROS burst is triggered by RaxX-sY in *N. benthamiana*.** ROS burst and cumulative ROS production over 90 min in leaf discs of *N. benthamiana* transiently expressing Myc-XA21 (XA21) and treated with mock, RaxX-sY, or RaxX-Y (non-sulfated negative control). ROS was measured by a luminol-based chemiluminescence assay and presented as RLUs. Error bars represent SD of biological replicates (*n* = 3). Asterisk represents significant differences (**p* < 0.050, one-way ANOVA, Tukey’s test). This experiment was repeated three times with similar results.

**
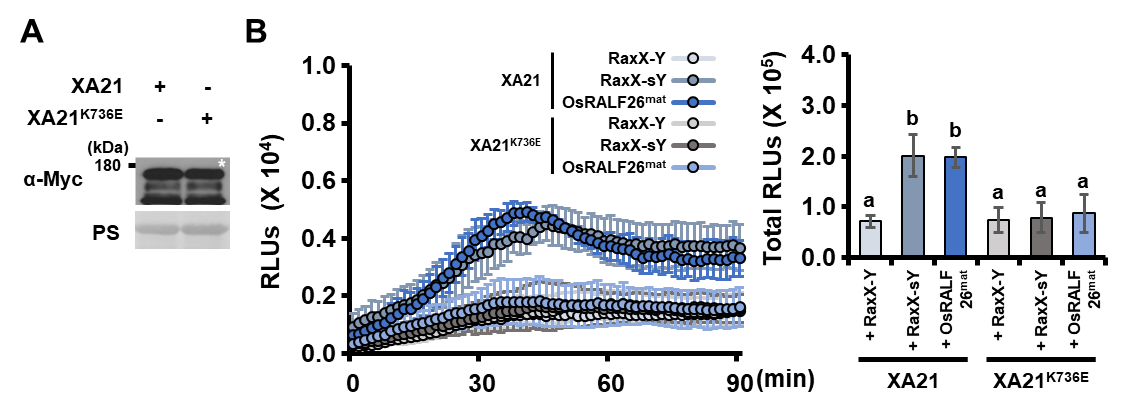
**

**Figure S3. XA21 kinase activity is required for OsRALF26-triggered ROS production in *N. benthamiana*. (A)** Transient expression of Myc-XA21 or kinase-dead Myc-XA21^K736E^ in *N. benthamiana* leaves via agroinfiltration. Protein accumulation was analyzed at 2 days after infiltration by immunoblotting using anti-Myc antibodies. Ponceau S (PS) staining is shown as a loading control. Asterisk (*) indicates bands corresponding to Myc-XA21 and XA21^K736E^. **(B)** ROS burst and cumulative ROS production over 90 min in leaf discs transiently expressing XA21 or XA21^K736E^ and treated with 1 μM of RaxX-Y, RaxX-sY, and OsRALF26^mat^. ROS was measured using a luminol-based chemiluminescence assay and presented as RLUs. Error bars represent SD of biological replicates (*n* = 3). Different letters indicate statistically significant differences (p < 0.05, one-way ANOVA followed by Tukey’s test). This experiment was repeated three times with similar results.

**
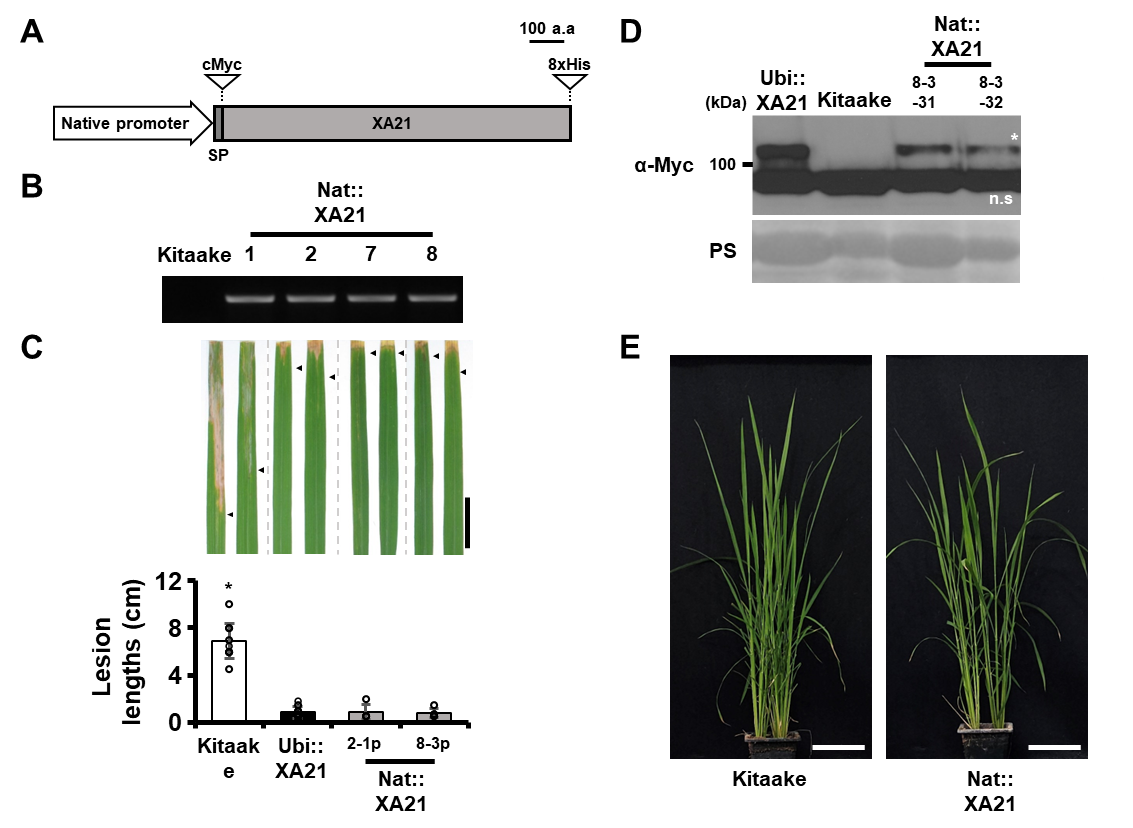
**

**Figure S4. Generation of transgenic rice plants expressing XA21 under its native promoter (Nat::XA21). (A)** Schematic representation of the Nat::XA21 construct. The *XA21* coding region (1025 amino acids) is driven by the native *XA21* promoter. A cMyc epitope tag was inserted immediately after the signal peptide, and an 8×His tag was fused to the C-terminus. SP, signal peptide. **(B)** Genotyping of Nat::XA21 lines using by PCR with the *Pmi* selection marker. **(C)** Lesion length measured 14 days after *Xoo*^WT^ inoculation in Kitaake, Ubi::XA21, and Nat::XA21 plants (T_2_; progeny of lines 2 and 8). Arrowheads indicate the bottom boundary of the lesion on each leaf. Scale bar, 2 cm. Error bars represent SD of biological replicates (*n* > 4). Asterisk represents significant differences (***p* < 0.001, one-way ANOVA, Tukey’s test). **(D)** Western blot analysis of Myc-XA21 in Kitaake, Nat::XA21 plants (T_2_; progeny of line 8), and Ubi::XA21 (positive control) using anti-Myc (α-Myc) antibody. Total proteins were extracted from leaves. Ponceau S (PS) staining was used as a loading control. Myc-XA21, ~116.58 kDa. Asterisk (*) indicates Myc-XA21; n.s., non-specific band. **(E)** Representative images of 8-week-old Kitaake and Nat::XA21 plants. Scale bar, 10 cm. All experiments were repeated at least three times with similar results.


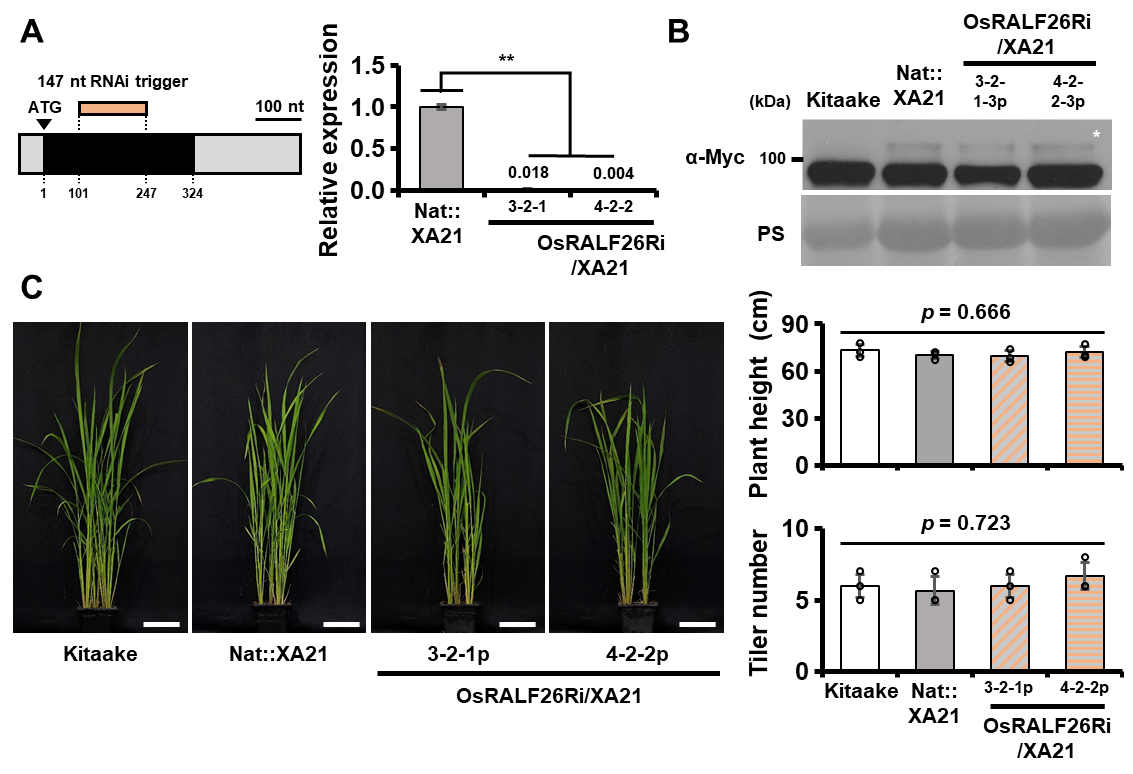


**Figure S5. Generation of *OsRALF26*-silenced transgenic rice in the Nat::XA21 background (OsRALF26Ri/XA21). (A)** Schematic diagram of the OsRALF26 RNAi target site and relative expression levels of *OsRALF26* in Nat::XA21 and OsRALF26Ri/XA21 plants. Nat::XA21, transgenic Kitaake expressing XA21; OsRALF26Ri/XA21, transgenic rice silencing *OsRALF26* in the background XA21 plants (T_2_; progeny of lines 3 and 4). Black box, coding sequence; gray boxes, untranslated region; orange box, 147 nt-length of RNAi target site (nucleotides 101–247 of OsRALF26 coding sequence). Error bars represent SD of technical replicates (*n* = 3). **(B)** Western blot analysis of Myc-XA21 in Kitaake, Nat::XA21, and OsRALF26Ri/XA21 (T_4_; progeny of lines 3 and 4) using anti-Myc (α-Myc) antibody. Total proteins were extracted from leaves. Ponceau S (PS) staining was used as a loading control. Myc-XA21, ~116.58 kDa. Asterisk (*) indicates Myc-XA21. **(C)** Agronomic traits of OsRALF26Ri/XA21 plants at eight weeks, including plant height and tiller number. In the photograph, from left to right: Kitaake, Nat::XA21, and OsRALF26Ri/XA21 (T_3_; progeny of lines 3 and 4). Scale bar, 10 cm. Error bars represent SD of biological replicates (*n* = 3). Asterisk represents significant differences (***p* < 0.001, one-way ANOVA, Tukey’s test). All experiments were repeated at least three times with similar results.

**
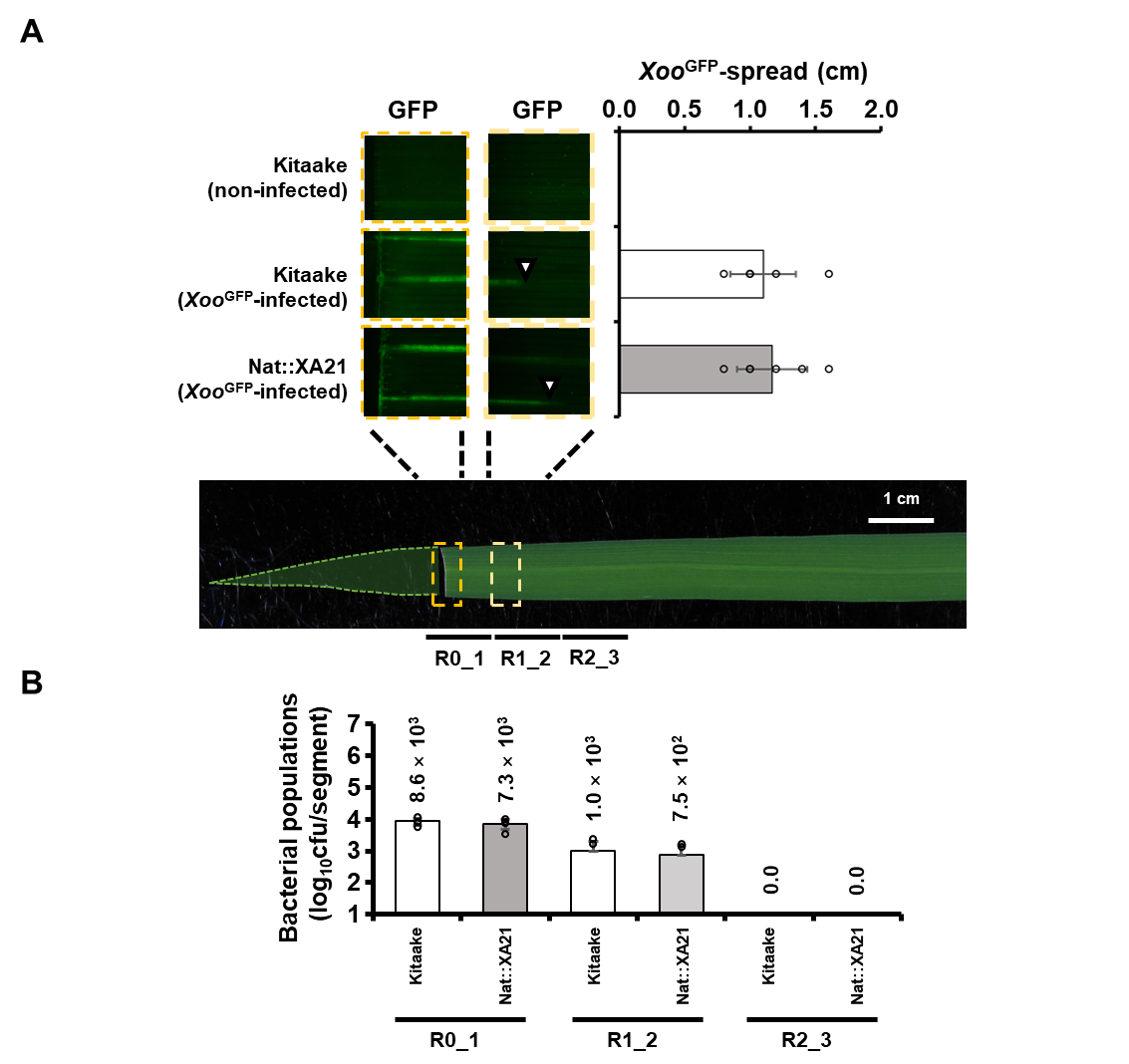
**

**Figure S6. *Xoo* movement and XA21-mediated defense response in distal rice leaf regions. (A)** Bacterial movement after *Xoo^GFP^* inoculation in 8-week-old Kitaake and Nat::XA21 rice leaves. GFP fluorescence was examined in regions R0_1 (0–1 cm), R1_2 (1–2 cm), and R2_3 (2–3 cm) from the inoculation site at 3 days after inoculation. Left panels show representative GFP fluorescence images; arrowheads mark the limit of detectable signal. Right panel quantifies the distance of *Xoo*^GFP^ (GFP-labeling *Xoo*) spread from the inoculation site. Error bars represent SD of biological replicates (*n* = 6). **(B)** Bacterial populations (log_10_cfu/segment) measured in corresponding segments, R0_1 (0–1 cm), R1_2 (1–2 cm), and R2_3 (2–3 cm), from Kitaake and Nat::XA21 rice leaves. Error bars represent SD of biological replicates (*n* = 3). All experiments were repeated at least three times with similar results.

**
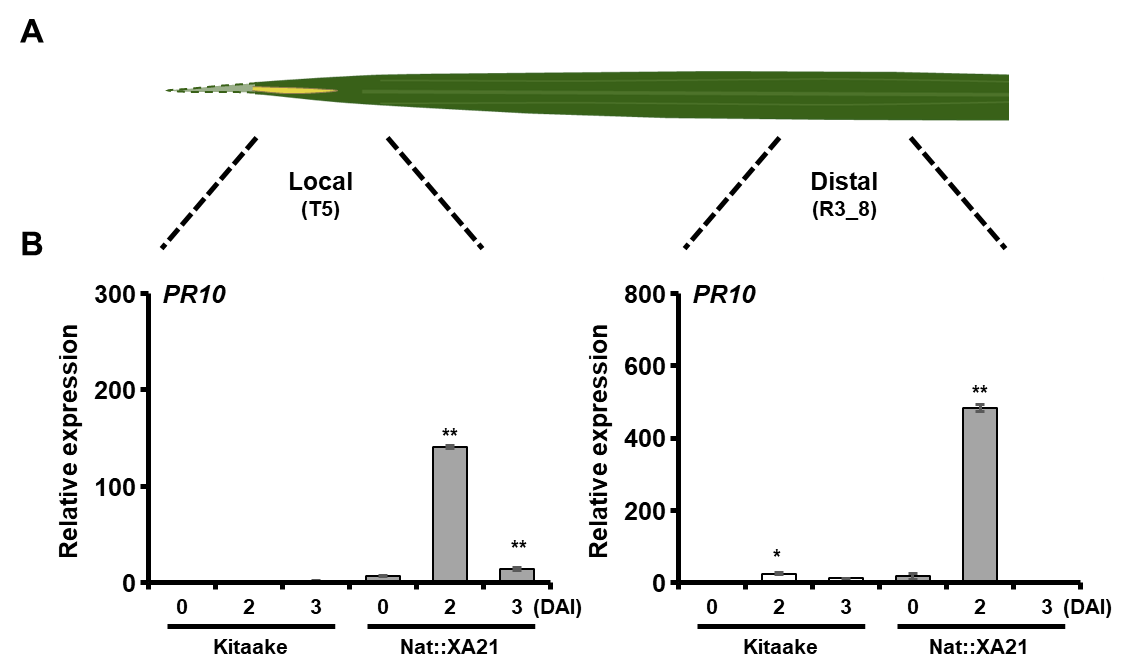
**

**Figure S7. Induction of *PR10* in local and distal leaf regions after *Xoo* inoculation. (A)** Representative rice leaf showing sampling sites. T5 indicates the local region (0–5 cm from the leaf tip, including the *Xoo*^WT^ inoculation site), and R3_8 represents the distal region (3–8 cm toward the leaf base from T5). **(B)** *PR10* expression levels in T5 and R3_8 of Kitaake and Nat::XA21 plants at 0, 2, and 3 days after *Xoo*^WT^ inoculation. Error bars indicate SD of technical replicates (*n* = 3). Asterisks indicate significant differences compared (**p* < 0.050, ***p* < 0.001, one-way ANOVA, Tukey’s test). All experiments were repeated three times with similar results.


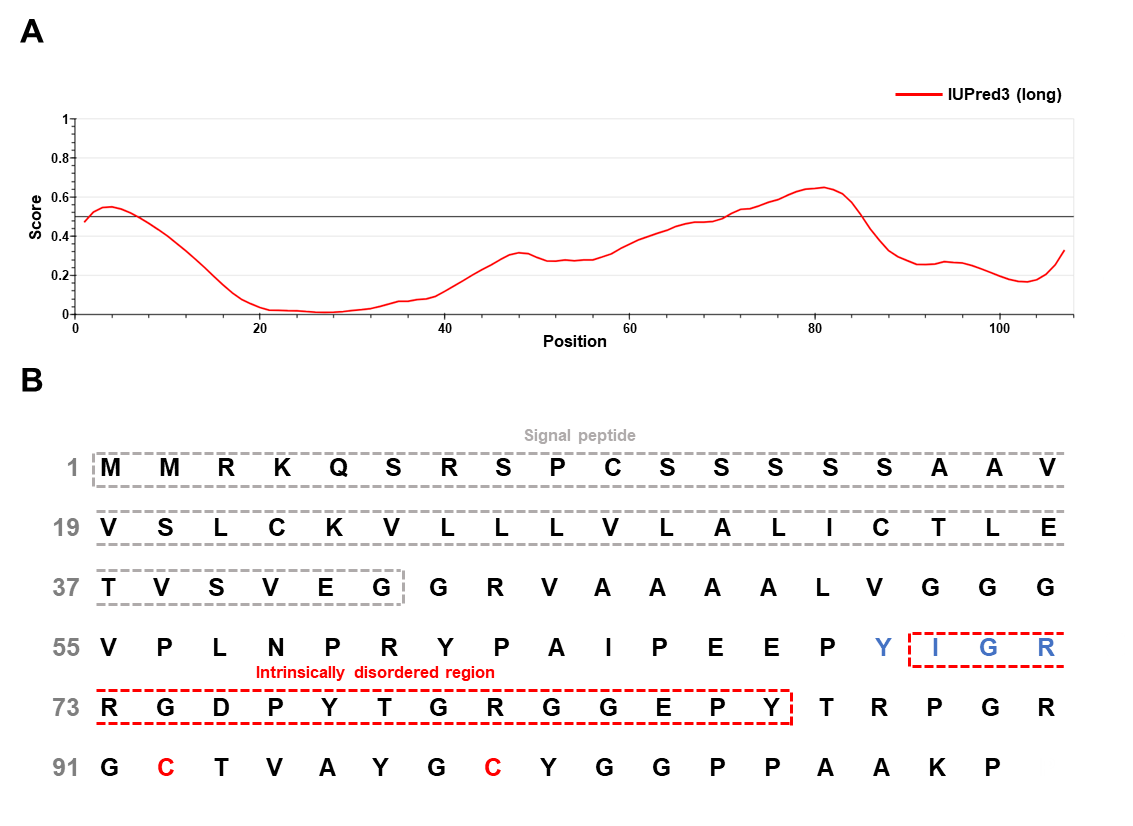


**Figure S8. Predicted intrinsically disordered regions of OsRALF26. (A)** Prediction of intrinsically disordered regions in OsRALF26 using IUPred3 (https://iupred3.elte.hu/). Red line: IUPred3 score; values >0.5 indicate an intrinsically disordered region. **(B)** Amino acid sequence of OsRALF26 showing the signal peptide and a predicted intrinsically disordered region. The N-terminal signal peptide (residues 1–42) is outlined in grey. The intrinsically disordered region (residues 70–85), predicted using IUPred3, is outlined in red. The OsFLR1-interacting YIGR motif (Kwon et al., 2024) is highlighted in blue. Red letters indicate two conserved cysteine residues (C92 and C98) implicated in disulfide bond formation and structural stabilization of RALF peptides.


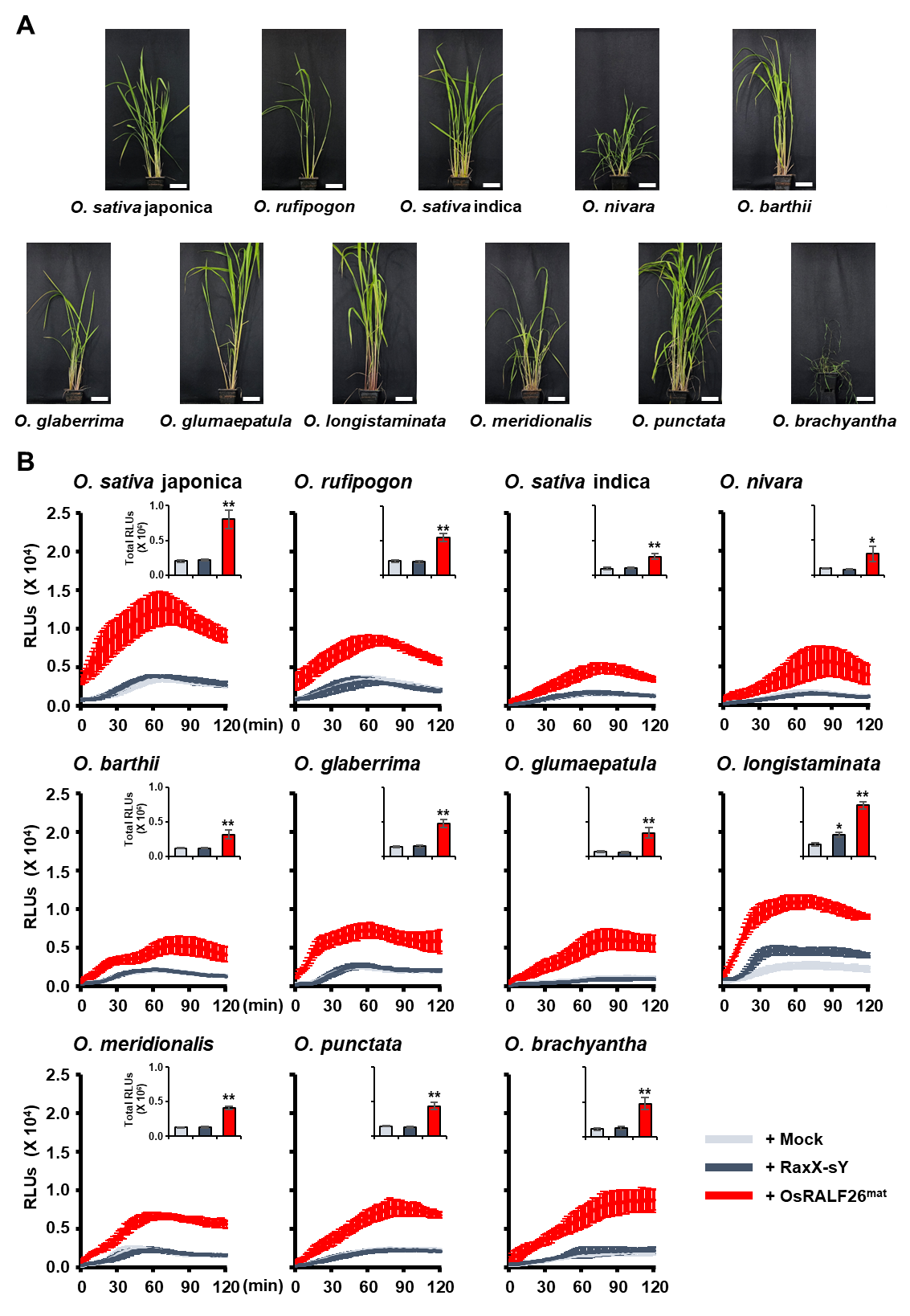


**Figure S9. Evolutionary distribution of immune responsiveness to OsRALF26 and RaxX in wild rice species. (A)** Representative 10-week-old plants of 11 wild rice species. Seeds were obtained from the International Rice Research Institute (IRRI) (see Table S1 for details). **(B)** ROS burst and cumulative ROS production over 120 min in leaf discs of 10-week-old plants from the 11 wild rice species treated with 1 μM RaxX21-sY or OsRALF26^mat^. ROS was measured by a luminol-based chemiluminescence assay and presented as accumulated RLUs. Error bars represent SD of biological replicates (*n* = 4). Asterisks represent significant differences (**p* < 0.050, and ***p* < 0.001, one-way ANOVA, Tukey’s test). This experiment was repeated three times with similar results.

**Table S1.** **List of *Oryza* accessions obtained from the International Rice Research Institute (IRRI).**

| **Accession No.** | **Accession Name** | **Species** |
| --- | --- | --- |
| IRGC 117274 | NIPPONBARE | *Oryza sativa* japonica |
| IRGC 100657 | TWC 1-12-2 | *Oryza rufipogon (Griff.)* |
| IRGC 84895 | IR8 | *Oryza sativa* indica |
| IRGC 100897 | W 106 | *Oryza nivara (Sharma et Shastry)* |
| IRGC 104084 | W 1616 | *Oryza barthii (A. Chev)* |
| IRGC 96717 | CG 14 | *Oryza glaberrima (Steud.)* |
| IRGC 100971 | W1191 | *Oryza glumaepatula (Steud.)* |
| IRGC 86537 | AU 96-002 | *Oryza meridionalis (Ng)* |
| IRGC 105690 | EAINR 20272 | *Oryza punctata (Kotschy ex Steud.)* |
| IRGC 101232 | W 1402 | *Oryza brachyantha (A. chev et Roehr.)* |
| IRGC 110404 | IRGC 110404 | *Oryza longistaminata (A. chev. et Roehr)* |

**Table S2. Primers used in this study.**

| **Primer name** | **Sequence (5′-3′)** | **Comments** | **Reference** |
| --- | --- | --- | --- |
| OsRALF26_RNAi_R | CGCCTCCCCGACCTGTGTA | R primer to clone OsRALF26_RNAi into pENTR | In this study |
| OsRALF26_101-120_F | CCCTGGAAACTGTCTCAGTA | F primer for RT-qPCR analysis of *OsRALF26* and to clone OsRALF26_ RNAi into pENTR | (Kwon et al., 2024) |
| OsRALF26_FL_R | TCATGGTTTGGCAGCTGGAG | R primer for RT-qPCR analysis of *OsRALF26* | (Kwon et al., 2024) |
| CACC_XA21_ECD_F | CACCATGGAGCAAAAGCTGATTTCTGA | F primer to clone XA21^ECD^ into pENTR | In this study |
| XA21_ECDwithSTOP_R | TTATAGAACTGGGAAATGTT | R primer to clone XA21^ECD^ into pENTR | In this study |
| XA21_KD_K736E_F | GTTGCAGTGGAAGTACTAAAGCTTGAAAATCC | F primers for site-directed mutagenesis of XA21 (K736E) | In this study |
| XA21_KD_K736E_R | ATGATCTTGGATATTAAGCTTTC | R primers for site-directed mutagenesis of XA21 (K736E) | In this study |
| pmi-1 | CCGCCGGAGATATCGTTTCACTG | F primer for genotyping of Nat::XA21 | (Qiu et al., 2015) |
| pmi-2 | CACGGTTCACCCTGCTGGCTATC | R primer for genotyping of Nat::21 | (Qiu et al., 2015) |
| PR-2(Gns5)-F | AAGATTGTTCTGAGAAGAGATCGATCGA | F primer for RT-qPCR analysis of *PR2* | (Kwon et al., 2024) |
| PR-2(Gns5)-R | GCTACGCGAAAATAGGTCTGGTAAACTT | R primer for RT-qPCR analysis of *PR2* | (Kwon et al., 2024) |
| PR10_qPCR_F | GCAGTGGTGTGATCAGTAGG | F primer for RT-qPCR analysis of *PR10* | (Kwon et al., 2024) |
| PR10_qPCR_R | TTGAGCTTCATGGTGGTGAC | R primer for RT-qPCR analysis of *PR10* | (Kwon et al., 2024) |
| OsActin-Q1 | TCGGCTCTGAATGTACCTCCTA | F primer for RT-qPCR analysis of *OsActin* | (Pruitt et al., 2015) |
| OsActin-Q2 | CACTTGAGTAAAGACTGTCACTTG | R primer for RT-qPCR analysis of *OsActin* | (Pruitt et al., 2015) |
